# Genome-wide analysis of expansin and aquaporin genes and their association with auxin-related metabolites during flower opening in *Lonicera macranthoides*

**DOI:** 10.3389/fpls.2026.1769953

**Published:** 2026-04-22

**Authors:** Zhong Chen, Junpeng Qi, Wei Zhuo, Yuqi Wang, Li Liu, Sheng’E Lu, Han Wang, Jibin Zhu, Fengming Ren

**Affiliations:** 1 Chongqing Institute of Medicinal Plant Cultivation, Chongqing University of Chinese Medicine, Chongqing, China; 2 Bio-resource Research and Utilization Joint Key Laboratory of Sichuan and Chongqing, Chongqing Institute of Medicinal Plant Cultivation, Chongqing, China; 3 Chongqing Traditional Chinese Medicine Hospital, Chongqing, China; 4 School of Chinese Materia Medica, Chongqing University of Chinese Medicine, Bishan, Chongqing, China

**Keywords:** aquaporin, auxin, expansin, flower-opening, *L. macranthoides*

## Abstract

**Introduction:**

In flowering plants, expansins and aquaporins function coordinately to promote cell expansion, thereby driving flower opening. *Lonicera macranthoides* is a widely used medicinal plant rich in chlorogenic acid (CGA) and saponins. Its flowers have high medicinal value; however, the contents of these bioactive compounds decrease after flower opening, and open flowers are prone to abscission. To date, the expansin and aquaporin gene families in *L. macranthoides* have not been systematically studied, and their roles in flower opening remain unclear.

**Methods:**

Expansin and aquaporin genes were identified from a chromosome-level genome using hidden Markov models. Phylogenetic relationships, chromosomal localization, protein sequence features, gene structures, and cis-regulatory elements were further analyzed. Gene expression patterns were examined using transcriptomic data and quantitative real-time PCR (qRT-PCR), and auxin-related metabolites were quantified by liquid chromatography–tandem mass spectrometry (LC–MS/MS).

**Results and discussion:**

Here, 34 expansin and 43 aquaporin genes were identified and systematically characterized. Expression analyses showed that *LmEXPA26* (EXPA subfamily) and *LmPIP2-2* (PIP2 subfamily) exhibited expression patterns that closely matched flower developmental progression during opening in *L. macranthoides*, with peak expression in S4 flowers of the flowering wild type (WT) but much lower expression in the bud-type cultivar ‘Yulei 1’. Promoter analysis revealed abundant MYB-, bZIP-, and ARF-binding sites, together with multiple ABA-, JA-, Auxin-, and ethylene-responsive elements in the promoters of expansin and aquaporin genes. Transcriptome analysis revealed significant enrichment of auxin-responsive genes in WT S4 flowers. Auxin-related metabolite profiling showed higher accumulation of MEIAA, a reversibly inactive auxin form convertible to IAA, in WT S4 flowers, whereas irreversibly inactivated OxIAA accumulated predominantly in S4 flowers of ‘Yulei 1’. Based on these results, the present study suggests that enhanced expression of expansins and aquaporins, especially *LmEXPA26* and *LmPIP2-2*, may be associated with the flower-opening process in *L. macranthoides*, which is likely primarily influenced by MEIAA-mediated auxin activation, although other hormones may also contribute. This study provides new insights into the molecular basis of flower opening in *L. macranthoides*.

## Introduction

*Lonicera macranthoides* is an important medicinal plant widely used in traditional Chinese medicine. Its high content of chlorogenic acids and various saponins contributes to its anti-inflammatory, antiviral, and antioxidant activities (Tang et al., 2021; Gao et al., 2023; Yin et al., 2023). The flower is the primary medicinal part of *L. macranthoides*, and its commercial value is closely linked to flower quality and yield, making the flowering process a key biological trait. However, the flowering period of *L. macranthoides* (Wild-type) is short, and open flowers abscise easily, accompanied by a rapid decline in active compounds (Pan et al., 2021; Zeng et al., 2025). Consequently, bud-type cultivars that do not open, such as ‘Yulei 1’ and ‘Xianglei 3’, are commonly used in production. Although abundant flower opening can increase productivity, excessive or asynchronous flower opening may reduce uniformity and complicate cultivation management. Therefore, understanding the mechanisms underlying flower opening is essential for improving the medicinal and agronomic performance of this species.

Flower opening requires coordinated cell wall remodeling, turgor pressure changes, and water transport (van Doorn and Kamdee, 2014). These processes are regulated by hormonal signals and environmental cues through multiple genetic pathways (Cho et al., 2017; Izawa, 2021). Many studies have shown that members of the expansin and aquaporin gene families play key roles in these flower-opening processes (Li et al., 2021; Guan et al., 2025). Expansins are nonenzymatic proteins that loosen plant cell walls by disrupting hydrogen bonding between cellulose microfibrils and hemicelluloses, thereby enabling turgor-driven cell expansion without hydrolytic activity (Wang et al., 2024b). They typically consist of 250–275 amino acids and contain two conserved domains: a six-stranded double-psi β-barrel (DPBB) and a family-63 carbohydrate-binding module (CBM63/Pollen_allerg). The expansin superfamily is generally classified into four subfamilies—EXPA, EXPB, EXLA, and EXLB (Lv et al., 2020). Many members are highly expressed in petals, where they promote petal cell expansion and facilitate flower opening. For example, *OfEXPA2* and *OfEXPA4* in *Osmanthus fragrans* (Miao et al., 2024), and *LiEXPA10*, *LiEXPA19*, *LiEXLA1*, and *LiEXLA2* in *Lagerstroemia indica* (Zhang et al., 2025), have been shown to participate in these processes.

Aquaporins (AQPs) are integral membrane proteins that facilitate the rapid and regulated transport of water and small neutral molecules across the plasma membrane (Li et al., 2025b). Plant AQPs are classified into seven phylogenetic subfamilies (PIPs, TIPs, NIPs, SIPs, XIPs, GIPs, and HIPs), although most higher plants retain only PIPs, TIPs, NIPs, and SIPs (Uppuluri et al., 2021). AQPs typically contain six transmembrane helices and two conserved NPA motifs that form the central water pore, along with an ar/R selectivity filter and additional diagnostic residues that determine substrate specificity and distinguish water-conducting aquaporins from aquaglyceroporins (Maurel et al., 2008; Uppuluri et al., 2021). In the context of flowering, aquaporins promote rapid water influx into petal cells, thereby increasing turgor pressure to support petal expansion and flower opening (Li et al., 2021). For example, the petal-highly expressed *GsPIP2;2* in *Gentiana scabra* plays an important role in regulating reversible flower opening (Nemoto et al., 2022). In *Rosa hybrida*, reducing the expression of *Rh-PIP2;1* through virus-induced gene silencing (VIGS) results in incomplete flower opening (Ma et al., 2008).

However, the expansin and aquaporin gene families in *L. macranthoides* have not been systematically characterized, and their roles in the flowering process remain unclear. Similarly, the molecular mechanisms regulating flower opening in *L. macranthoides* are largely unexplored. To address these gaps, this study aims to characterize the expansin and aquaporin gene families in *L. macranthoides* and investigate their potential involvement in flower opening. By integrating gene family identification, sequence analysis, transcriptome and gene expression profiling, and auxin-related metabolite measurements, we seek to clarify how these genes contribute to the flower-opening process. The findings will provide new insights into the regulatory mechanisms of flower opening in *L. macranthoides* and support future research on genetic improvement and cultivation strategies.

## Materials and methods

### Identification of expansin and aquaporin genes in *L. macranthoides*

The assembled genome of *L. macranthoides* was reported in the previous study (Yin et al., 2023). The genome assembly and annotation files were obtained from Professor Xiaojian Yin, who is currently affiliated with the Northeast Institute of Geography and Agroecology, Chinese Academy of Sciences. Expansin and aquaporin protein sequences of *Arabidopsis thaliana* were downloaded from TAIR10 (http://www.arabidopsis.org/). Candidate expansin proteins were identified using hidden Markov model (HMM) searches performed with HMMER (version 3.4; http://hmmer.org/) using default parameters. The Pfam HMM profiles PF01357 (Expansin C-terminal domain/PHL pollen allergen) and PF03330 (Expansin N-terminal domain/DPBB) were used as queries to search the *L. macranthoides* protein sequences, with an E-value threshold of 1e−5. Proteins containing both PF01357 and PF03330 domains were further validated using InterProScan (http://www.ebi.ac.uk/interpro/) and retained as the final expansin dataset. Similarly, candidate aquaporin proteins were identified using the Pfam HMM profile PF00230 with the same HMMER search parameters. The identified sequences were subsequently confirmed using InterProScan to obtain the final aquaporin dataset. Protein physicochemical properties were analyzed using ExPASy ProtParam (https://web.expasy.org/protparam/).

### Phylogenetic tree construction of expansin and aquaporin proteins

Phylogenetic analyses of the expansin and aquaporin protein families were conducted using the protein sequences identified in *L. macranthoides* together with homologous sequences from *Arabidopsis thaliana*. Multiple sequence alignments were generated using MAFFT with default parameters (Katoh et al., 2019). The aligned sequences were then used to construct maximum-likelihood phylogenetic trees using IQ-TREE with the best-fit substitution model LG+I+G (Lanfear et al., 2020). Branch support was evaluated using 1,000 bootstrap replicates. The resulting phylogenetic trees were visualized and edited using iTOL (http://itol.embl.de).

### Chromosomal distribution, gene structure, and conserved motif analysis of expansin and aquaporin families

Chromosomal locations and gene structures of expansin and aquaporin genes in *L. macranthoides* were obtained from the assembled genome and GFF3 annotation files, and visualized using TBtools (Chen et al., 2020). Conserved motifs were identified with MEME Suite using default parameters (http://meme-suite.org/tools/meme), with a maximum of 10 motifs, and the resulting patterns were also visualized in TBtools for comparative analysis among family members.

### Promoter analysis of expansin and aquaporin genes

Promoter sequences of expansin and aquaporin genes in *L. macranthoides* were extracted combined the assembled genome and GFF3 annotation files. For each gene, approximately 2 kb upstream of the ATG start codon and 500 bp downstream were retrieved as putative promoter regions. AuxREs (auxin response elements) in the promoters of *LmEXPA26* and *LmPIP2–2* were manually identified. Other cis-acting regulatory elements were predicted using the PlantCARE database (http://bioinformatics.psb.ugent.be/webtools/plantcare/html/). All data were visualized using the ggplot2 package in R (https://ggplot2.tidyverse.org/).

### Plant materials, RNA-seq and qRT-PCR data analysis of expansin and aquaporin genes

Cultivar ‘Yulei 1’ and wild-type *L. macranthoides* plants were cultivated in the experimental field of the Chongqing Institute of Medicinal Plant Cultivation (CQIMPC), located in Xiushan County, Chongqing, China (108°58′ E, 28°27′ N). All plants were identified by Researcher Zhengyu Liu. Roots, stems, leaves, and flowers at developmental stages S3–S5 (as described previously by Zeng et al., 2025) from both materials were collected on June, 2025. The samples were immediately frozen in liquid nitrogen and stored at –80 °C until further use. Three independent biological replicates were sampled for each tissue.

For transcriptome sequencing, total RNA was extracted using a standard plant RNA extraction kit (Aidlab, Beijing, China), RNA purity and concentration were determined using a NanoDrop One spectrophotometer (Thermo Fisher Scientific, USA). Samples with A260/A280 ratios of approximately 2.0 and A260/A230 ratios greater than 2.0 were considered suitable for further analysis. RNA integrity was evaluated by agarose gel electrophoresis, revealing two clear and distinct bands corresponding to 28S and 18S rRNA. RNA detection, sequencing, and routine data processing were carried out by Majorbio (Shanghai, China) according to their standard procedures. Differentially expressed genes (DEGs) were identified using thresholds of false discovery rate (FDR) ≤ 0.05 and |log_2_(fold change)| ≥ 1. Raw RNA-sequencing (RNA-seq) data were deposited in the NCBI Sequence Read Archive (SRA) under accession number PRJNA1368365.

For qRT-PCR validation, first-strand cDNA was synthesized from 1 μg of total RNA using the PrimeScript RT reagent kit (Vazyme, Nanjing, China) following the manufacturer’s instructions. qRT-PCR assays were performed using SYBR Green Master Mix (Vazyme) on a CFX96 Real-Time PCR system (Bio-Rad, CA, USA) with gene-specific primers for expansin and aquaporin genes (**Supplementary Table 6**). Primer design was performed using Primer3 (v2.6.1, https://github.com/primer3-org/primer3/releases/tag/v2.6.1) with the following parameters: primer length of 18–25 bp (optimal 20 bp), melting temperature (Tm) of 59-62 °C (optimal 60 °C), GC content of 40-60%, and product size ranging from 160 to 220 bp. Primers with potential secondary structures, excessive GC content at the 3’ end, or long homopolymeric stretches were excluded. In addition, each primer pair was subjected to Primer-BLAST analysis (https://www.ncbi.nlm.nih.gov/tools/primer-blast/) against the coding sequence (CDS) FASTA file of *L. macranthoides* to ensure that each primer pair specifically matched only its intended target sequence. Moreover, the specificity of the selected primers was further confirmed by agarose gel electrophoresis and melting curve analysis. The amplification efficiencies of all 21 primer pairs ranged from 94.20% to 109.72%, which fall within the acceptable range of 90%-110% for qRT-PCR primers (**Supplementary Table 6**). The PCR conditions were as follows: an initial denaturation at 95 °C for 1 min, followed by 40 cycles of 95 °C for 20 s and 60 °C for 1 min. *LmUBQ* was used as the internal reference gene to normalize transcript levels. Each assay included three independent biological replicates. Relative expression levels were calculated using the 2^-ΔCt^ method as previously described (Livak and Schmittgen, 2001; Yu et al., 2024), where ΔCt was defined as Ct_target - Ct_reference. The mean values and standard errors (SE) were calculated for statistical analysis.

### Determination of auxin and auxin-related metabolite contents

Auxin and auxin-related metabolites were analyzed by MetWare (Wuhan, Hubei, China) using the AB Sciex QTRAP 6500 LC–MS/MS platform, as previously described (He et al., 2020; Yang et al., 2024). The methodological validation details have been provided in **Supplementary Table 15**. In addition, the precision and recovery of this LC–MS/MS method using internal standard quantification were previously validated by the analytical platform across various tissues and multiple plant species before the present analysis (He et al., 2020; Qin et al., 2023; Jing et al., 2024; Yang et al., 2024). The analytical procedure is briefly described as follows: fresh plant tissues were frozen in liquid nitrogen, ground into powder, and 50 mg of each sample was extracted with 1 mL methanol/water/formic acid (15:4:1, v/v/v) containing 10 μL of internal standards (100 ng/mL). After centrifugation, drying, re-dissolution in 100 μL of 80% methanol, and filtration, 2 μL of each extract was injected for LC–MS/MS analysis. Chromatographic separation was performed on an ExionLC™ AD UPLC system with a Waters ACQUITY HSS T3 C18 column using a water–acetonitrile gradient containing 0.04% acetic acid. Metabolites were detected and quantified on a QTRAP^®^ 6500+ mass spectrometer using scheduled MRM, and data were processed with Analyst 1.6.3 and MultiQuant 3.0.3.

## Results

### Identification and characterization of expansin and aquaporin gene families in *L. macranthoides*

To investigate the potential roles of expansin and aquaporin families during flower opening in *L. macranthoides*, we performed a comprehensive identification and analysis of both gene families. Based on the *L. macranthoides* genome, hidden Markov models (HMMs) PF01357 (Expansin C-terminal/PHL pollen allergen) and PF03330 (Expansin N-terminal/DPBB) were used to yield 42 candidate expansin protein sequences. These sequences were further validated using InterProScan, and only proteins containing both PF01357 and PF03330 domains were retained. A total of 34 expansin proteins were ultimately identified (**Table 1**; **Supplementary Table 1**, **S2**). Similarly, 43 aquaporin candidates were retrieved using HMM model PF00230. Domain confirmation was performed with InterProScan using major functional databases including Pfam, SMART, PRINTS, CDD, and PANTHER. Conserved NPA motifs, Ar/R filters, and Froger’s residues were also detected as typical features of aquaporins (**Supplementary Table 3**, **S4**). Therefore, all 43 aquaporin proteins were retained for downstream analyses (**Table 1**; **Supplementary Table 2**).

**Table 1:** Physicochemical properties of expansin and aquaporin families in *l. macranthoides*.

Protein name	Gene ID	Protein length (aa)	Isoelectric point	Molecular weight (kDa)	GRAVY	Instability index
Expansins						
LmEXPA4a	*EVM0014778*	256	9.4	27.86	0.001	32.9
LmEXPA4b	*EVM0020166*	259	9.6	28	-0.009	36.22
LmEXPA4c	*EVM0038444*	260	9.55	28.31	-0.053	26.4
LmEXPA4d	*EVM0015354*	252	9.21	27.42	-0.224	36.56
LmEXPA5a	*EVM0020560*	214	8.51	23.26	-0.13	26.21
LmEXPA5b	*EVM0033249*	457	9.19	49.66	-0.219	35.91
LmEXPA5c	*EVM0039279*	250	9.53	26.86	-0.113	42.08
LmEXPA6	*EVM0005558*	259	9.33	27.93	0.029	36.24
LmEXPA7	*EVM0027034*	540	8.1	60.05	-0.126	46.97
LmEXPA8a	*EVM0030793*	255	8.06	27.63	-0.197	27.54
LmEXPA8b	*EVM0005929*	252	8.06	26.96	-0.135	29.77
LmEXPA8c	*EVM0024296*	250	8.37	26.54	-0.035	25.73
LmEXPA11a	*EVM0006026*	253	9.33	27.56	-0.163	32.09
LmEXPA11b	*EVM0025931*	258	9.01	27.59	-0.037	26.82
LmEXPA12	*EVM0002705*	259	9.95	28.74	-0.094	40.54
LmEXPA13	*EVM0022745*	263	8.6	28.52	-0.056	34.06
LmEXPA14	*EVM0017574*	246	9.2	26.12	-0.032	32.94
LmEXPA15a	*EVM0007964*	242	9.45	26.51	-0.101	31.98
LmEXPA15b	*EVM0020761*	240	8.99	25.79	0.002	34.88
LmEXPA20	*EVM0035789*	256	8.67	28.29	-0.039	32.93
LmEXPA26	*EVM0000903*	268	9.42	29.42	-0.286	29.14
LmEXPA27	*EVM0023064*	261	8.96	28.27	-0.114	30.32
LmEXPB2a	*EVM0007937*	265	6.28	27.83	0.025	41.35
LmEXPB2b	*EVM0011066*	269	6.58	28.69	-0.044	40.67
LmEXPB3	*EVM0036479*	266	8.87	28.57	-0.011	33.7
LmEXPB5a	*EVM0036072*	261	8.5	28.29	0.108	24.66
LmEXPB5b	*EVM0014433*	226	8.51	24.39	0	22.61
LmEXPB5c	*EVM0020787*	261	8.65	28.23	0.114	26.16
LmEXLA2	*EVM0026112*	259	8.82	28.27	-0.126	28.96
LmEXLA4	*EVM0021118*	263	8.24	28.39	0.058	43.77
LmEXLA5	*EVM0000164*	257	6.28	28.18	-0.13	32.64
LmEXLA6	*EVM0016196*	256	4.49	28.03	-0.222	36.35
LmEXLB1a	*EVM0037794*	244	7.44	26.88	-0.04	26.53
LmEXLB1b	*EVM0034214*	243	8	27.34	-0.13	29.75
Aquaporins						
LmPIP1-1	*EVM0012175*	286	8.82	30.44	0.434	28.56
LmPIP1-2	*EVM0037103*	384	9.67	40.42	0.711	25.57
LmPIP1-3	*EVM0017892*	213	9.23	22.38	0.595	24.11
LmPIP1-4	*EVM0008003*	286	8.81	30.6	0.367	31.11
LmPIP1-5	*EVM0036603*	286	8.64	30.63	0.319	31.07
LmPIP2-1	*EVM0003215*	173	6.9	19.12	0.246	32.68
LmPIP2-2	*EVM0034581*	287	7.69	30.67	0.457	30.74
LmPIP2-3	*EVM0024450*	285	8.26	30.75	0.407	26.97
LmPIP2-4	*EVM0015953*	283	6.88	30.28	0.473	29.05
LmPIP2-5	*EVM0012913*	285	8.58	30.61	0.518	29.81
LmPIP2-7	*EVM0038078*	254	8.71	27.06	0.437	35.06
LmPIP2-8	*EVM0023048*	282	9.07	30.11	0.424	33.62
LmTIP1-1	*EVM0028212*	252	6.01	26.07	0.755	23.83
LmTIP1-3	*EVM0016034*	252	5.14	26.28	0.727	29.3
LmTIP1-4	*EVM0039017*	242	5.12	25.08	0.818	30.24
LmTIP1-5	*EVM0030680*	252	4.94	25.96	0.853	30.96
LmTIP1-6	*EVM0026939*	253	4.96	26.11	0.866	31.58
LmTIP2-1	*EVM0010501*	248	5.76	25.17	0.946	25.4
LmTIP2-2	*EVM0004254*	250	5.09	25.39	0.899	28.97
LmTIP2-3	*EVM0025357*	271	6.01	28.02	0.929	28.6
LmTIP3-1	*EVM0018571*	254	9.3	26.86	0.403	42.99
LmTIP3-2	*EVM0002993*	246	6.11	26.06	0.503	32.16
LmTIP4-1	*EVM0009909*	248	5.71	26.07	0.888	21.1
LmTIP5-1	*EVM0029342*	252	5.89	26.22	0.6	36.6
LmNIP2-1	*EVM0036827*	281	6.51	30.37	0.543	42.42
LmNIP3-1	*EVM0027627*	274	9.32	29.11	0.553	23.09
LmNIP3-2	*EVM0029384*	192	8.83	20.71	0.528	36.25
LmNIP3-3	*EVM0007177*	281	8.82	29.85	0.476	28.46
LmNIP4-1	*EVM0028991*	272	6.43	29.06	0.603	40.14
LmNIP4-2	*EVM0029508*	256	5.17	27.2	0.619	32.25
LmNIP4-3	*EVM0036367*	207	9.48	22.19	0.463	40.36
LmNIP5-1	*EVM0022122*	287	9.22	29.81	0.462	36.18
LmNIP5-2	*EVM0002298*	293	8.87	30.34	0.537	35.43
LmNIP6-1	*EVM0039643*	304	9.06	31.45	0.482	30.33
LmNIP6-2	*EVM0000995*	303	7.01	31.47	0.509	32.42
LmNIP7-1	*EVM0019995*	287	8.85	30.24	0.53	29.75
LmNIP7-2	*EVM0039433*	295	6.29	31.31	0.589	32.51
LmNIP7-3	*EVM0013280*	281	8.45	29.61	0.75	35.11
LmNIP7-4	*EVM0037069*	281	8.45	29.57	0.758	32.37
LmNIP7-5	*EVM0027398*	281	8.45	29.58	0.759	34.67
LmSIP1-1	*EVM0014276*	238	8.57	25.07	0.83	23.11
LmSIP1-2	*EVM0037017*	220	8.95	22.87	0.747	29.54
LmSIP2-1	*EVM0001160*	237	9.39	25.99	0.559	29.71

GRAVY (Grand Average of Hydropathy) represents the overall hydrophobicity of a protein, with positive values indicating hydrophobic proteins and negative values indicating hydrophilic proteins. The Instability Index reflects the *in vitro* stability of a protein, where values < 40 suggest a stable protein and values > 40 indicate an unstable protein.

**Figure 1:**
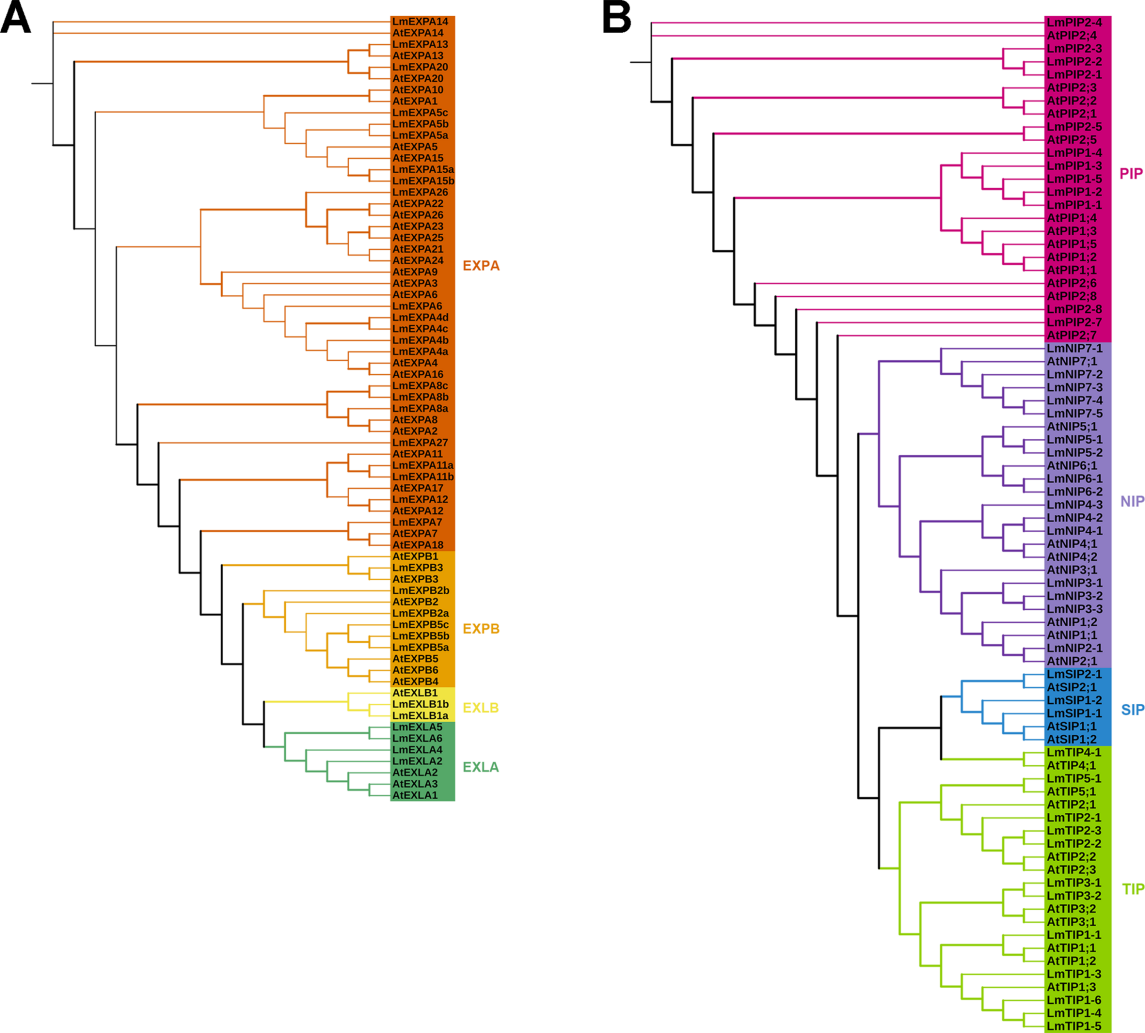
Phylogenetic analysis of expansin and aquaporin proteins in *L. macranthoides* and *A. thaliana*. Maximum-likelihood phylogenetic trees of expansin **(A)** and aquaporin **(B)** proteins were constructed using protein sequences from *L. macranthoides* together with homologs from *A. thaliana*. Bold branches indicate bootstrap support >70% based on 1,000 replicates.

To determine orthologous and paralogous relationships of *L. macranthoides* expansin and aquaporin genes, we obtained 35 expansin and 35 aquaporin protein sequences from *Arabidopsis thaliana* and constructed phylogenetic trees with the *L. macranthoides* sequences. Based on tree topology and *Arabidopsis* nomenclature (Johanson et al., 2001; Kende et al., 2004), 34 expansins were assigned to four subfamilies: 22 EXPA, 6 EXPB, 4 EXPLA, and 2 EXPLB. The 43 aquaporins were classified into 12 PIPs, 12 TIPs, 16 NIPs, and 3 SIPs (**Figures 1A, B**; **Table 1**). Comparison of NPA motifs, Ar/R filters, and Froger’s residues further showed that proteins with closer phylogenetic relationships exhibited higher sequence similarity in these conserved regions, supporting the reliability of the phylogenetic reconstruction.

Protein property analyses showed that most expansins were 240–270 aa in length, although LmEXPA5a and LmEXPB5b were shorter, while LmEXPA5b and LmEXPA7 were longer. Their isoelectric points predominantly ranged from 8.0 to 10.0. GRAVY values indicated that most expansins were hydrophilic, consistent with their cell wall localization, and instability index values suggested that most were predicted to be stable (**Table 1**). For aquaporins, protein lengths ranged from 173 to 384 aa, with most clustering around 250–280 aa. Their molecular weights were mainly 25–31 kDa, and isoelectric points generally ranged from 8.0 to 10.0. GRAVY values indicated strong hydrophobicity, consistent with membrane-associated proteins. Instability index analysis showed that most aquaporins were predicted to be stable. Notably, LmTIPs exhibited lower isoelectric points (4.94–6.11) than other aquaporin subfamilies, except LmTIP3-1 (9.3). Moreover, except for LmTIP3–1 and LmTIP3-2, LmTIPs showed GRAVY values greater than 0.7, mostly within 0.818–0.946. These properties clearly distinguished LmTIPs from other aquaporins and provide useful information for future purification of LmTIP proteins (**Table 1**).

### Chromosomal distribution and structural features of expansin and aquaporin families in *L. macranthoides*

**Figure 2:**
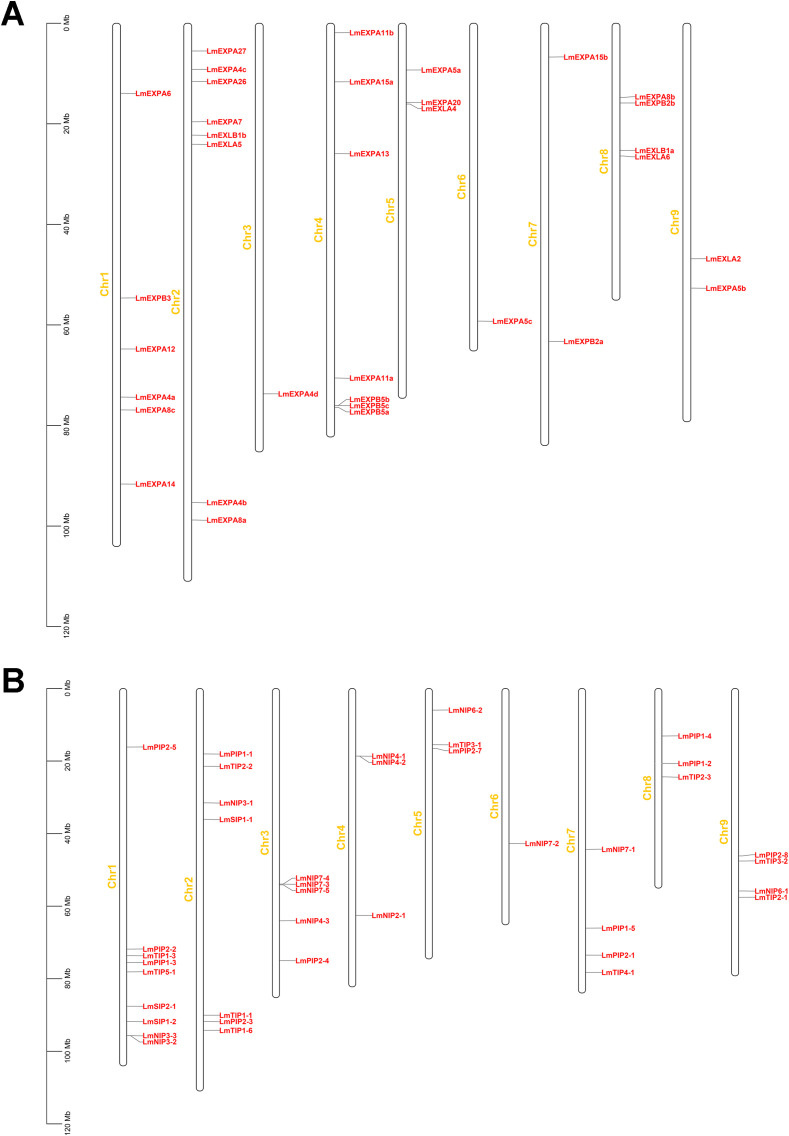
Locations of the expansin **(A)** and aquaporin **(B)** Families on 9 chromosomes of *L. macranthoides*. Four aquaporin genes were located on unplaced contigs rather than the nine assembled chromosomes (**Supplementary Table 5**).

The *L. macranthoides* genome contains nine chromosomes (Yin et al., 2023). Expansin genes were mainly distributed on chromosomes 1, 2, and 4, harboring 6, 8, and 7 expansin genes, respectively. Chromosomes 3 and 6 contained only one expansin gene each, while chromosomes 5, 7, and 9 carried 3, 2, and 2 genes, respectively; chromosome 8 contained 4 expansin genes (**Figure 2A**; **Supplementary Table 5**). For aquaporins, the majority were located on chromosomes 1, 2, and 3, with 9, 7, and 5 genes, respectively. Chromosome 6 contained only one aquaporin gene. In addition, chromosomes 4, 5, and 8 harbored three aquaporin genes each, while chromosomes 7 and 9 contained four aquaporin genes each (**Figure 2B**). Four aquaporin genes—*LmTIP1-4*, *LmNIP5-1*, *LmTIP1-5*, and *LmNIP5-2*—were not anchored to any of the nine chromosomes but were instead located on four unplaced contigs. The positions of these genes and the lengths of the corresponding contigs are provided in **Supplementary Table 5**.

**Figure 3:**
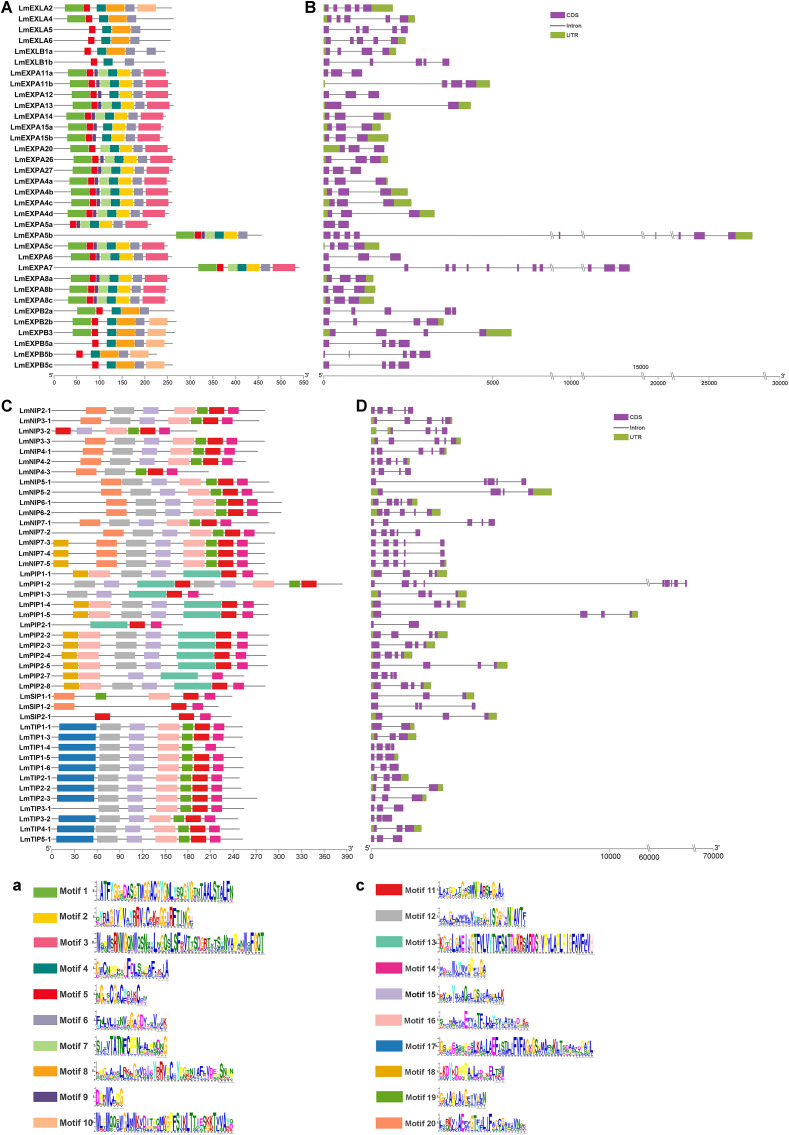
Conserved motifs of expansin and aquaporin proteins and the exon–intron structures of their encoding genes in *L. macranthoides*. **(A)** Conserved protein motifs of expansins identified using MEME, corresponding to the motifs shown in panel **(a)**. **(B)** Exon–intron structures of expansin genes. **(C)** Conserved protein motifs of aquaporins identified using MEME, corresponding to the motifs shown in panel **(c)**. **(D)** Exon–intron structures of aquaporin genes. In **(B)** and **(D)**, colored boxes represent exons and dark gray lines indicate introns. Among the exonic regions, purple boxes represent coding sequences (CDS), whereas green boxes denote untranslated regions (UTRs). The scales in **(A)** and **(C)** indicate protein length in amino acids (aa), whereas the scales in **(B)** and **(D)** indicate gene length in nucleotides (bp), double slashes (\\) indicate omitted sequences.

Conserved protein motifs represent key structural features of the expansin and aquaporin families. Subfamily-specific motifs serve as distinct sequence signatures and may contribute to functional divergence among subfamilies. In **Figure 3A**, all expansin proteins contained motif 4, motif 5, and motif 6, suggesting that these motifs represent conserved domains characteristic of the expansin family. The LmEXPA subclass uniquely possessed motif 2, motif 3, motif 7, and motif 9. Motif 8 was present in LmEXPB, LmEXLA, and LmEXLB, while motif 10 occurred only in LmEXPB and LmEXLA. Motif 1 was found exclusively in LmEXPA and LmEXLA proteins (**Figure 3A**). Gene structure analysis showed that the coding regions of most *LmEXPA* genes contained three exons, except *LmEXPA13*, *LmEXPA5a*, *LmEXPA5b*, and *LmEXPA7*. All coding regions of *LmEXLA* genes contained five exons, whereas those of *LmEXPB* and *LmEXLB* genes contained four or five exons (**Figure 3B**).

For aquaporins, motif 16, motif 11, and motif 14 were conserved across all subfamilies (**Figure 3C**). LmPIPs contained the unique motif 13, while LmTIPs uniquely harbored motif 17. Motif 18 appeared in LmPIPs and LmNIPs, whereas LmNIPs and LmSIPs both contained motif 20. Motif 19 was absent only in LmPIPs, and motif 12 and motif 15 were absent only in LmSIPs. Regarding gene structure, the coding regions of 13 of the 16 *LmNIPs* contained five exons, except *LmNIP3-2*, *LmNIP4-3*, and *LmNIP5-2*. Among *LmPIPs*, the coding regions of 10 of the 12 genes contained four exons, except *LmPIP1–2* and *LmPIP2-1*. For *LmTIPs*, the coding regions of 10 of the 12 genes contained three exons, while *LmTIP1–1* had two exons and *LmTIP1–4* had four. Among the *LmSIPs*, the coding regions of *LmSIP1–1* and *LmSIP2–1* contained three exons, whereas *LmSIP1–2* contained four exons (**Figure 3D**). Both the motif patterns and gene structural characteristics were consistent with the results in **Figure 1** and **Table 1**, further supporting the classification of expansin and aquaporin subfamilies in *L. macranthoides*.

### Expression patterns of expansin and aquaporin genes in *L. macranthoides*

**Figure 4:**
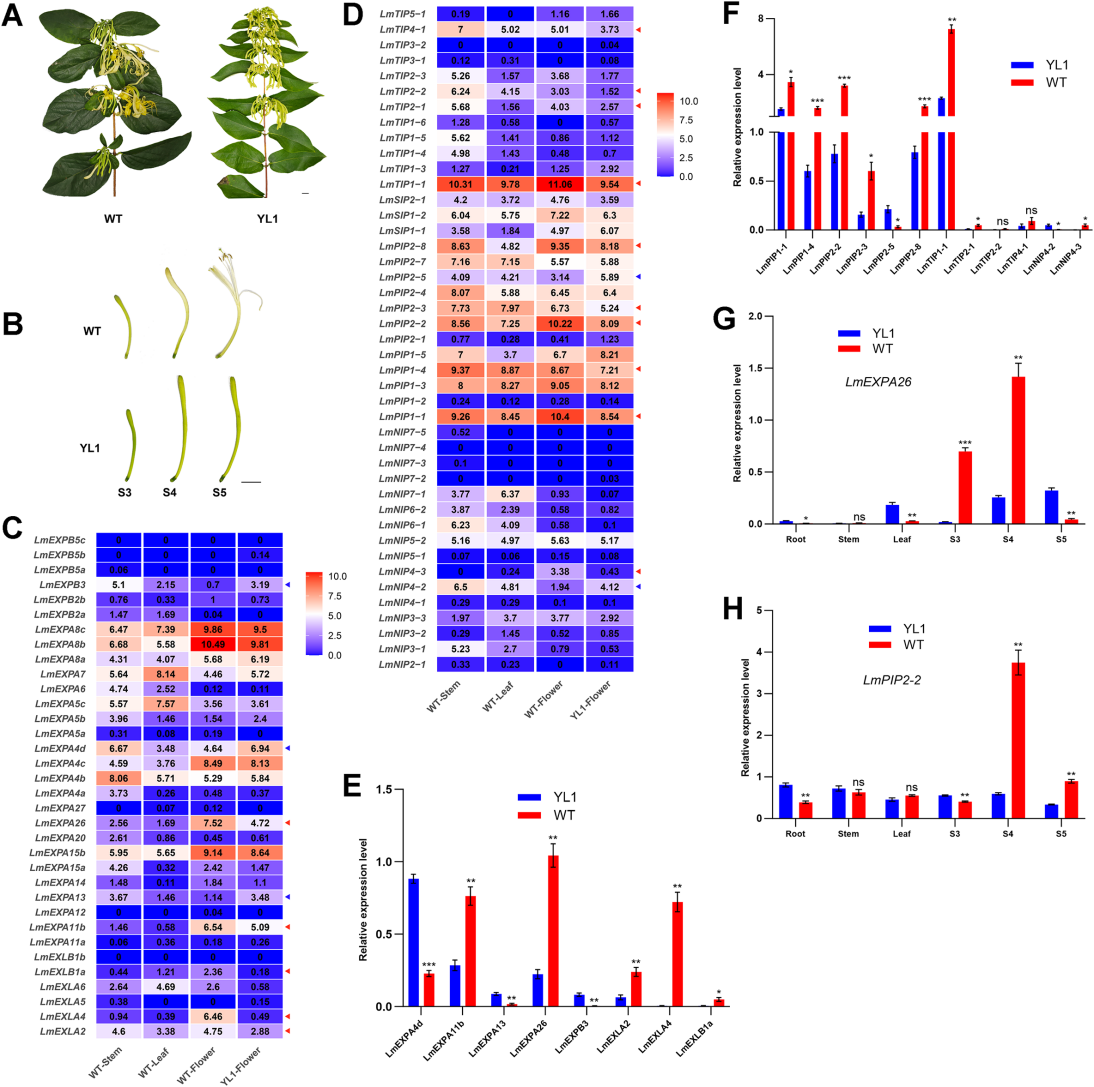
RNA-seq and qRT-PCR analyses of expansin and aquaporin genes in WT and ‘Yulei 1’. **(A)** Flowering branches of WT and ‘Yulei 1’. Bars = 1 cm. **(B)** Developmental stages S3, S4, and S5 of flowers in WT and ‘Yulei 1’. Bars = 1 cm. **(C)** RNA-seq expression profiles of expansin genes in WT stems, WT leaves, WT S4 flowers, and ‘Yulei 1’ S4 flowers. Values are shown as log_2_(TPM + 1). **(D)** RNA-seq expression profiles of aquaporin genes in WT stems, WT leaves, WT S4 flowers, and ‘Yulei 1’ S4 flowers. Values are shown as log_2_(TPM + 1). **(E)** qRT-PCR validation of differentially expressed expansin genes in S4 flowers of WT and ‘Yulei 1’. **(F)** qRT-PCR validation of differentially expressed aquaporin genes in S4 flowers of WT and ‘Yulei 1’. **(G)** qRT-PCR analysis of *LmEXPA26* expression in stems, leaves, and S3, S4, and S5 stage flowers of WT and ‘Yulei 1’. **(H)** qRT-PCR analysis of *LmPIP2–2* expression in stems, leaves, and S3, S4, and S5 stage flowers of WT and ‘Yulei 1’. WT, wild type of *L. macranthoides*. YL1, cultivar ‘Yulei 1’ of *L. macranthoides*. Red and blue arrows indicate significantly up-regulated and down-regulated genes in the RNA-seq analysis, respectively, TPM indicates transcripts per million mapped reads. All experiments were performed with three biological replicates. Error bar indicates standard error of the difference, * indicates p < 0.05, ** indicates p < 0.01, *** indicates p < 0.001, ns indicates no significant difference between YL1 and WT (p ≥ 0.05).

*L. macranthoides* exhibits two major phenotypes: a bud-type form represented by the cultivar ‘Yulei 1’ (not flowering open) and the wild type (flowering open). The bud-type ‘Yulei 1’ is a cultivar obtained from a wild-type mutant and propagated via cutting (**Figure 4A**). Therefore, ‘Yulei 1’ was used as the control and compared with the flower-opening wild type to understand the flower opening process in *L. macranthoides*. Following the developmental staging system described by Zeng et al. (2025), the flower-opening process of both ‘Yulei 1’ and the wild type (WT) was divided into three stages: S3, S4, and S5. At the S5 stage, the corolla of the wild type (WT) was fully open, whereas the corolla of ‘Yulei 1’ remained closed and failed to open, maintaining a bud-like state (**Figure 4B**).

RNA-seq analysis was performed using stems and leaves of WT, and S4-stage flowers of both WT and ‘Yulei 1’. When Genes with log_2_(TPM + 1) values < 1 in both WT and ‘Yulei 1’ flowers were filtered out, a total of 13 expansin genes showed higher expression in WT S4 flowers than in ‘Yulei 1’, while 8 expansin genes showed lower expression. In addition, *LmEXPA8c*, *LmEXPA8b*, *LmEXPA4d*, and *LmEXPA4b* were highly expressed in stems, whereas *LmEXPA8c*, *LmEXPA7*, and *LmEXPA5c* showed higher expression in leaves (**Figure 4C**). For aquaporin genes, 17 members were more highly expressed in WT S4 flowers than in ‘Yulei 1’, whereas nine exhibited lower expression. Several aquaporin genes, including *LmTIP4-1*, *LmTIP1-1*, *LmPIP2-8*, *LmPIP2-7*, *LmPIP2-4*, *LmPIP2-3*, *LmPIP2-2*, *LmPIP1-5*, *LmPIP1-4*, *LmPIP1-3*, and *LmPIP1-1*, were highly expressed in stems, with *LmTIP1–1* showing the highest expression, followed by *LmPIP1–4* and *LmPIP1-1*. Many genes also showed high expression in leaves, again with *LmTIP1–1* exhibiting the highest expression and *LmPIP1-4*, *LmPIP1-3*, and *LmPIP1–1* following (**Figure 4D**).

Differential expression analysis of the expansin and aquaporin gene families in S4-stage flowers of WT and ‘Yulei 1’ was performed with significance thresholds of FDR ≤ 0.05 and |log_2_(fold change)| ≥ 1. Compare to ‘Yulei 1’ S4 flowers, five expansin genes (*LmEXPA26*, *LmEXPA11b*, *LmEXLB1a*, *LmEXLA4*, and *LmEXLA2*) were significantly upregulated in WT S4 flowers, while three expansin genes (*LmEXPB3*, *LmEXPA4d*, and *LmEXPA13*) were significantly downregulated. For aquaporins, 10 genes (*LmTIP4-1*, *LmTIP2-2*, *LmTIP2-1*, *LmTIP1-1*, *LmPIP2-8*, *LmPIP2-3*, *LmPIP2-2*, *LmPIP1-4*, *LmPIP1-1*, and *LmNIP4-3*) were significantly upregulated, while two genes (*LmPIP2–5* and *LmNIP4-2*) were significantly downregulated (**Figure 4C** and D). Overall, more expansin and aquaporin genes were upregulated than downregulated in WT S4 flowers, and the magnitude of upregulation was greater than that of downregulation.

qRT-PCR validation of expansin genes with significant differential expression in WT and ‘Yulei 1’ S4 flowers confirmed the trends observed in the RNA-seq data, with *LmEXPA26* showing the highest expression level in WT S4 flowers (**Figure 4E**; **Supplementary Table 6**, **S7**). Similarly, qRT-PCR validation of the significantly differentially expressed aquaporin genes showed that *LmTIP2–2* and *LmTIP4–1* did not exhibit significant differences between WT and ‘Yulei 1’ S4 flowers, possibly due to their low expression levels in both genotypes. The expression trends of all other genes were consistent with the RNA-seq results, with *LmTIP1–1* being the most highly expressed gene in WT S4 flowers and *LmPIP2–2* showing the largest fold-change difference (4.39-fold) between WT and ‘Yulei 1’ S4 flowers (**Figure 4F**; **Supplementary Table 6**, **S7**).

To further investigate key candidate genes, *LmEXPA26* and *LmPIP2-2*, which exhibited high expression in S4 flowers of *L. macranthoides* and the greatest fold changes between WT and ‘Yulei 1’, were selected for expression validation in the roots, stems, leaves, and flowers at the S3, S4, and S5 stages of both genotypes. For *LmEXPA26*, its expression in WT flowers increased from S3 to S4, reaching the highest level at S4, where it was significantly higher than that in ‘Yulei 1’ at the same stage, and then decreased from S4 to S5. In ‘Yulei 1’ flowers, *LmEXPA26* expression gradually increased from S3 to S5, although its expression remained much lower than that in WT at the corresponding stages, except at S5. In roots and stems, *LmEXPA26* expression was low in both genotypes, whereas in leaves its expression was higher in ‘Yulei 1’ than in WT (**Figure 4G**; **Supplementary Table 6**, **S7**).

Similar to *LmEXPA26*, *LmPIP2–2* expression in WT flowers also increased from S3 to S4 and peaked at S4, where it was significantly higher than in ‘Yulei 1’, before decreasing from S4 to S5. However, in ‘Yulei 1’ flowers, *LmPIP2–2* expression showed a continuous decline from S3 to S5. *LmPIP2–2* expression was generally low in roots, stems, and leaves of both genotypes, and its expression in WT flowers at S4 was markedly higher than in any other tissue (**Figure 4H**; **Supplementary Table 6**, **S7**). Collectively, these results indicate that *LmEXPA26* and *LmPIP2–2* expression patterns correspond closely with flower-opening progression in WT from S3 to S5. In contrast, ‘Yulei 1’ flowers do not undergo normal morphological transition during these stages (aside from changes in flower size), and their gene expression patterns lack consistent developmental trends (**Figures 4B, G, H**). These findings suggest that the dynamic expression of *LmEXPA26* and *LmPIP2–2* may be associated with the flower-opening process in WT.

### Promoter analysis of expansin and aquaporin genes in *L. macranthoides*

**Figure 5:**
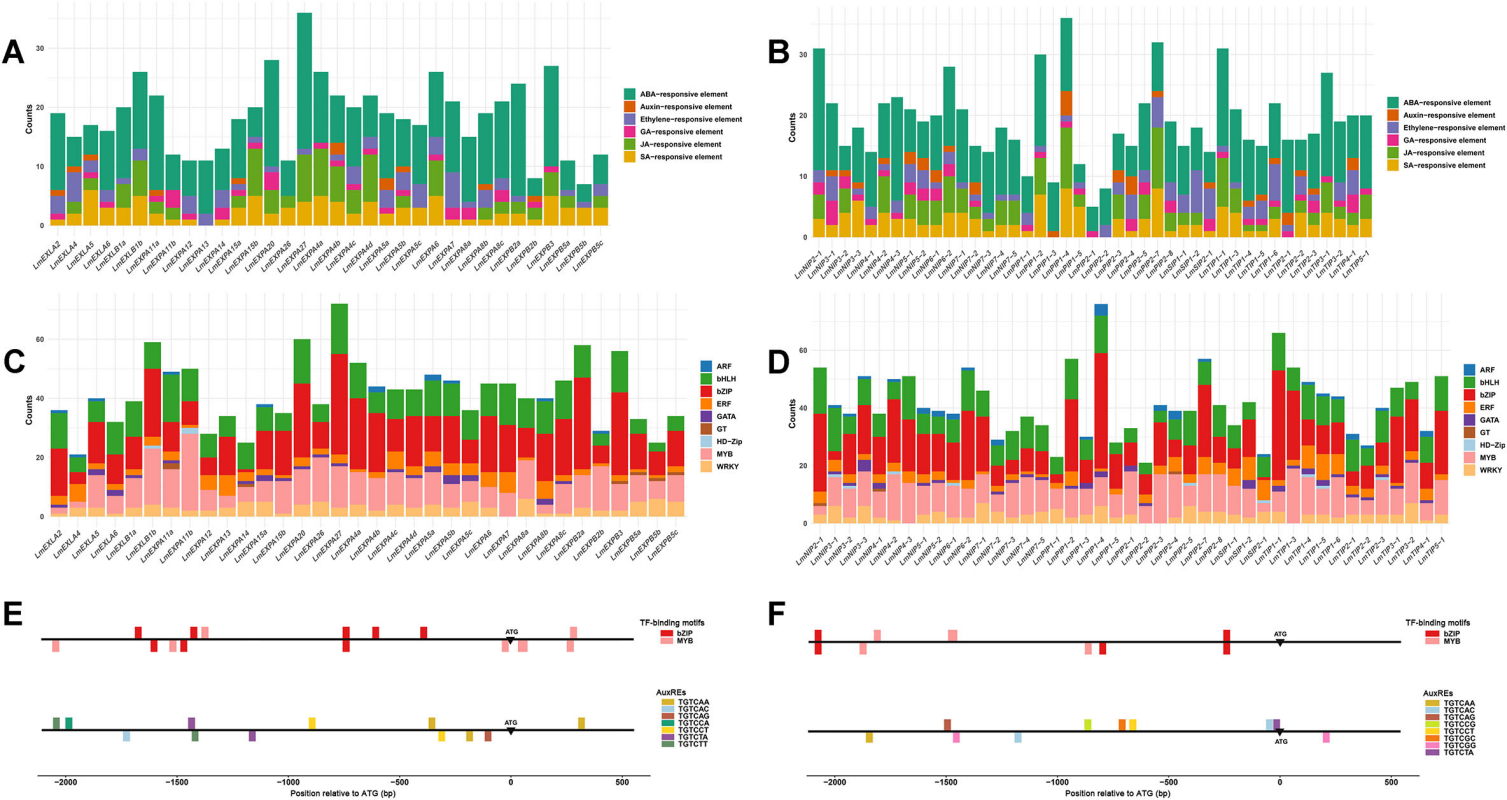
Predicted hormone-responsive elements and transcription factor binding sites in the promoters of expansin and aquaporin genes in *L. macranthoides*. **(A)** Predicted hormone-responsive elements in expansin gene promoters. **(B)** Predicted hormone-responsive elements in aquaporin gene promoters. **(C)** Predicted transcription factor binding sites in expansin gene promoters. **(D)** Predicted transcription factor binding sites in aquaporin gene promoters. **(E)** and **(F)** Distribution of TF-binding motifs and AuxREs (auxin response elements) in the promoters of *LmEXPA26* and *LmPIP2-2*, respectively. TF, transcription factor. The black line represents the promoter sequence. TF-binding motifs and AuxREs above the line are located on the positive DNA strand, whereas those below the line are located on the negative strand. Motifs upstream of the ATG are indicated by negative values, while those downstream of the ATG are indicated by positive values. The positive strand is oriented from 5′ to 3′ from left to right.

To investigate the reasons for the differential expression of expansin and aquaporin genes in *L. macranthoides*, 2.5 kb genomic regions spanning approximately 2 kb upstream and 0.5 kb downstream of the ATG start codon were extracted for promoter analysis. The results showed that the promoters of expansin and aquaporin genes contain numerous ABA-responsive, JA-responsive, ethylene-responsive, and SA-responsive elements. Specifically, the promoter of *LmEXPA26* is enriched in ABA-, JA-, and SA-responsive elements, whereas the promoter of *LmPIP2–2* mainly contains ABA- and ethylene-responsive elements (**Figures 5A, B**; **Supplementary Table 8**, **S9**).

Furthermore, we analyzed the binding sites for transcription factor families associated with plant growth and development, including ARF, ERF, GATA, MYB, WRKY, bHLH, bZIP, HD-Zip, and GT. The promoters of expansin and aquaporin genes were found to contain numerous binding sites for bHLH, bZIP, MYB, WRKY, and ERF transcription factors (**Figures 5C, D**; **Supplementary Table 8**, **S9**). The expansin subfamily EXPA and the aquaporin subfamily PIP2 play important roles in petal growth and development (Lu et al., 2024; Miao et al., 2024). Among these genes, *LmEXPA26* and *LmPIP2–2* showed the highest expression levels in S4 flowers of *L. macranthoides*. Analysis of their promoter regions revealed that MYB and bZIP transcription factor-binding sites were the most and second most abundant TF-binding motifs (**Figures 5E, F**; **Supplementary Table 8**, **S9, S10**), respectively. Previous studies have reported that auxin plays an important role in regulating petal growth and development (Ke et al., 2018; Chen et al., 2023). The DNA-binding element of auxin response factors (ARFs) in the auxin signaling pathway is the AuxRE motif (TGTCNN) (Boer et al., 2014; Lieberman-Lazarovich et al., 2019). Through manual inspection, multiple AuxREs were also identified in the promoters of *LmEXPA26* and *LmPIP2-2*. These results indicate that the expansin and aquaporin genes in *L. macranthoides* can be regulated by multiple hormone signals and transcription factors.

### Transcriptome analysis and auxin-related metabolite profiling in WT and ‘Yulei 1’ Flowers

**Figure 6:**
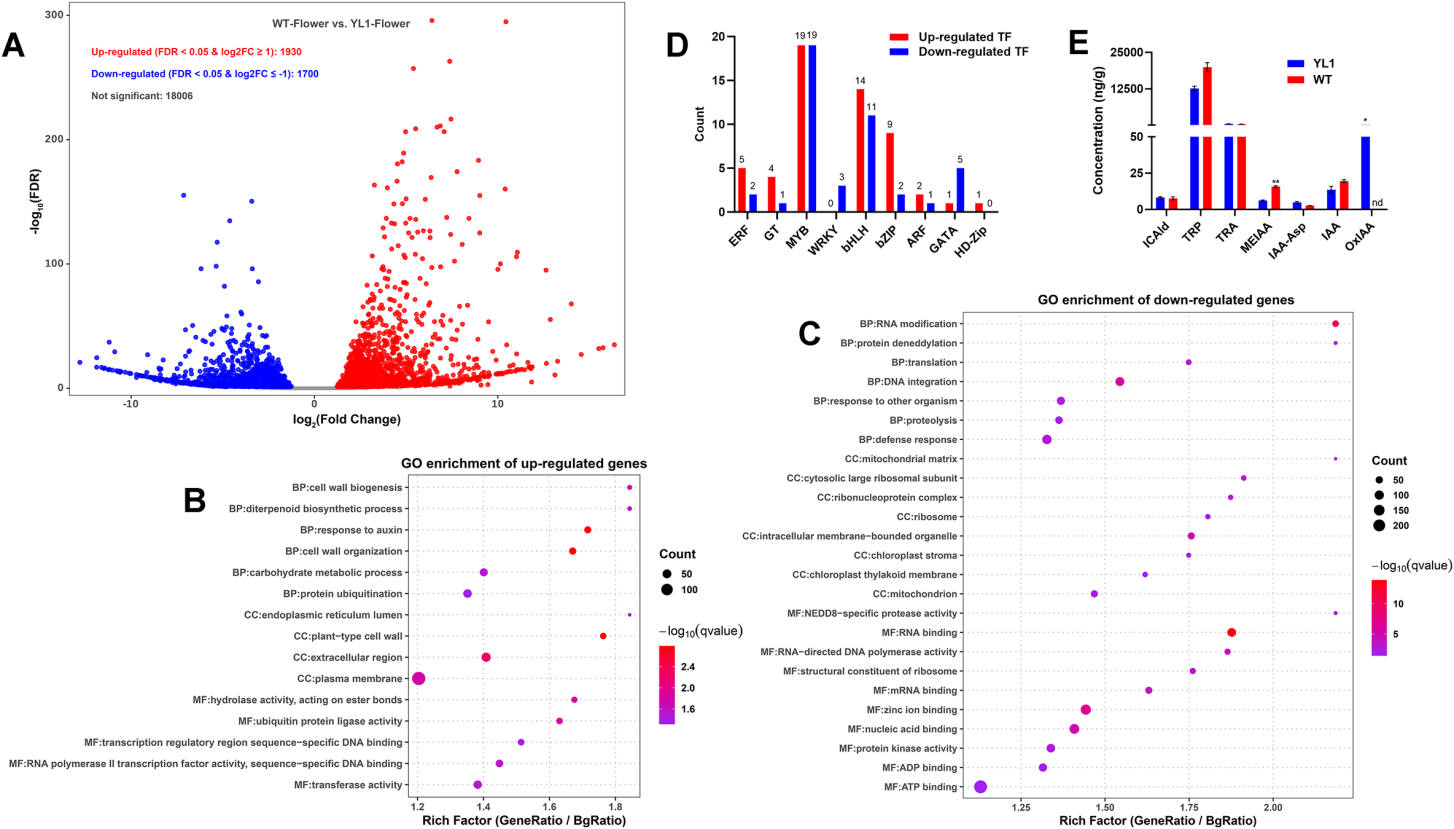
RNA-seq analysis and auxin-related metabolite content in S4 flowers of WT and ‘Yulei 1’. **(A)** Differentially expressed genes (DEGs) identified in S4 flowers of WT and ‘Yulei 1’. GO enrichment analysis of upregulated genes **(B)** and downregulated genes **(C)**, The rich factor represents the ratio of gene ratio to background ratio (BgRatio). **(D)** Transcription factors (TFs) identified among the DEGs. **(E)** Quantification of auxin-related metabolites in S4 flowers of WT and ‘Yulei 1’. All experiments were performed with three biological replicates, error bar indicates standard error of the difference, * indicates p < 0.05, ** indicates p < 0.01, except for MEIAA and OxIAA, no significant differences were observed in the other auxin-related metabolites between S4 flowers of WT and ‘Yulei 1’, nd, not detected.

To further investigate why the flower-opening process occurred only in WT but not in ‘Yulei 1’, we performed transcriptome sequencing of WT and ‘Yulei 1’ flowers at the S4 stage. With the threshold set to a false discovery rate (FDR) ≤ 0.05 and |log_2_(fold change)| ≥ 1, 1,930 genes were upregulated and 1,700 genes were downregulated in WT S4 flowers compared with ‘Yulei 1’ S4 flowers (**Figure 6A**; **Supplementary Table 11**). GO (Gene Ontology) enrichment analysis of the differentially expressed genes (DEGs) showed that, among the upregulated genes, those classified in the Biological Process (BP) category were mainly associated with cell-wall organization and auxin response. Genes in the Cellular Component (CC) category were enriched in terms related to the plasma membrane, cell wall, and extracellular region. Genes in the Molecular Function (MF) category were enriched in functions such as DNA binding, hydrolase activity, and transferase activity (**Figure 6B**; **Supplementary Table 12**).

Among the downregulated genes, most were associated with transcription, translation, protein degradation, and energy metabolism. Notably, genes in the CC category were predominantly related to chloroplast-associated processes (**Figure 6C**; **Supplementary Table 13**). These results are consistent with the flower phenotypes of WT and ‘Yulei 1’ at the S4 stage: WT flowers appeared whiter than ‘Yulei 1’, suggesting lower chloroplast abundance in WT S4 flowers. The downregulation of chloroplast-related genes in WT S4 flowers agrees with this phenotype. In addition, S4 is the stage immediately preceding flower opening (S5) in WT; therefore, compared to ‘Yulei 1’ S4 flowers, the strong upregulation of genes involved in cell-wall modification and auxin response in WT S4 flowers may play important roles in driving the flower-opening process. We also analyzed transcription factors (TFs) among the upregulated and downregulated DEGs, including ARF, ERF, GATA, MYB, WRKY, bHLH, bZIP, HD-Zip, and GT families. TF families with more upregulated than downregulated genes included ERF, GT, bHLH, bZIP, ARF, and HD-Zip. Among them, the bZIP family showed the highest ratio of upregulated to downregulated genes (4.5:1). The numbers of up- and downregulated MYB genes were similar. WRKY and GATA had more downregulated than upregulated genes (**Figure 6D**; **Supplementary Table 14**).

Based on the GO results (**Figure 6B**), auxin-related genes were the only hormone-related genes significantly upregulated in WT S4 flowers. Therefore, we further analyzed the levels of auxin-related metabolites in WT and ‘Yulei 1’ S4 flowers. Seven metabolites were detected, including TRP (L-tryptophan), TRA (tryptamine), MEIAA (methyl indole-3-acetate), ICAld (indole-3-carboxaldehyde), IAA-Asp (indole-3-acetyl-L-aspartic acid), IAA (indole-3-acetic acid), and OxIAA (2-oxindole-3-acetic acid). Only MEIAA and OxIAA showed significant differences between S4 flowers of WT and ‘Yulei 1’. MEIAA levels were significantly higher in WT S4 flowers (15.75 ng/g) than in ‘Yulei 1’ S4 flowers (6.19 ng/g). In contrast, OxIAA was not detected in WT S4 flowers but reached 124.75 ng/g in ‘Yulei 1’ S4 flowers (**Figure 6E**; **Supplementary Table 15**). We also measured auxin-related metabolites in WT S5 flowers. MEIAA levels decreased to 5.17 ng/g, similar to the level in ‘Yulei 1’ S4 flowers. Conversely, OxIAA increased sharply in WT S5 flowers, reaching 141.14 ng/g, even higher than the level in ‘Yulei 1’ S4 flowers (**Supplementary Table 15**). These data indicate that the high MEIAA content at the S4 stage may be an important factor enabling flower opening in WT of *L. macranthoides*.

### A proposed molecular model for flower opening in *L. macranthoides*

**Figure 7:**
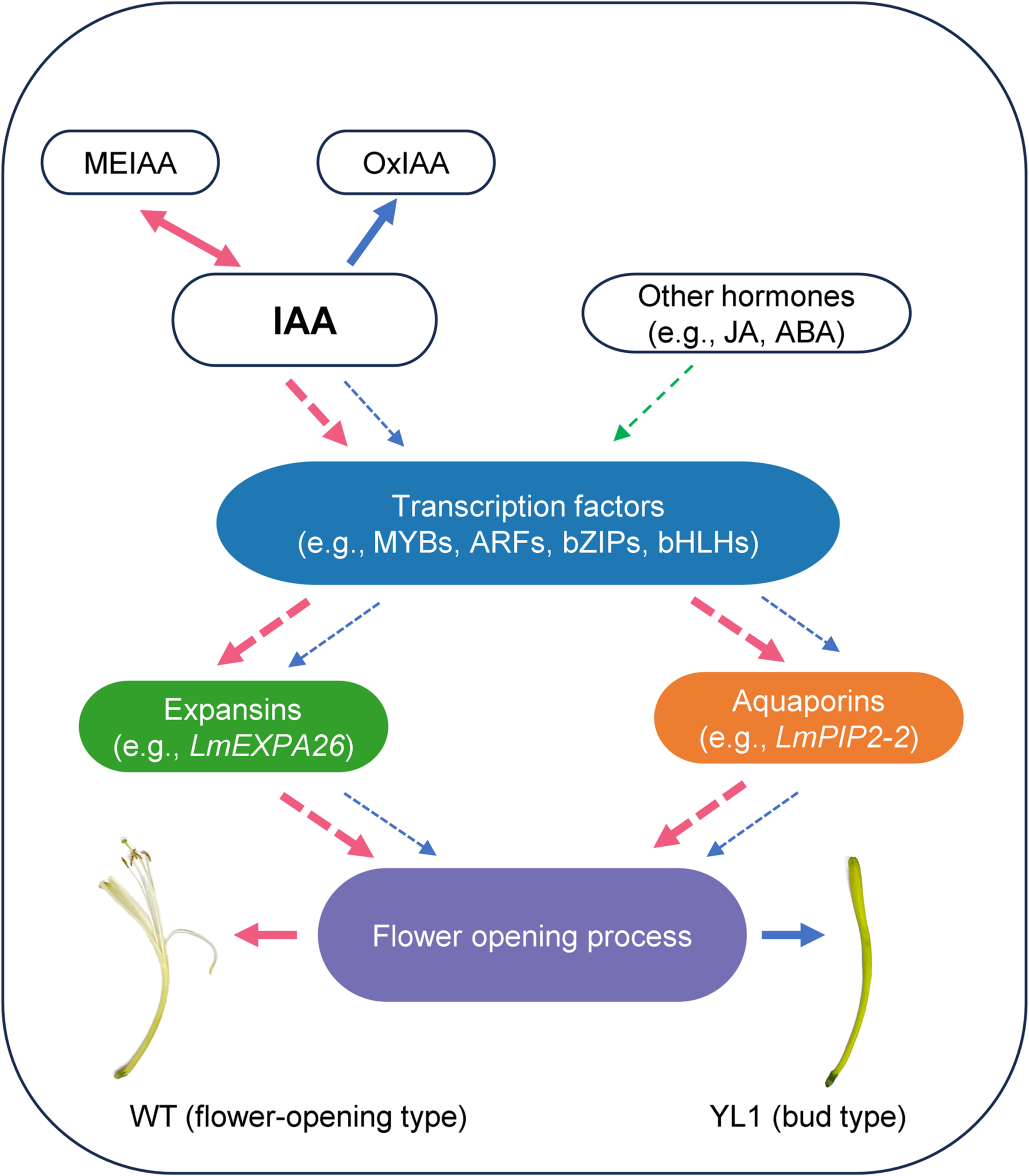
Schematic representation of the putative molecular model underlying flower opening in *L. macranthoides*. Red arrows indicate the direction of auxin-related metabolite changes and gene regulation in WT flowers, whereas blue arrows indicate the corresponding direction in ‘Yulei 1’ flowers. Green arrows represent the influence of other hormones. Arrow thickness reflects the relative strength of metabolite flux and gene regulatory activity.

As shown in **Figure 7**, based on cis-element analysis of promoters, gene expression data, and auxin-related metabolite profiling, we inferred a putative model for flower opening in *L. macranthoides*. In WT flowers, abundant MEIAA may be rapidly and reversibly converted into active IAA, which may promote the expression of transcription factors such as MYBs and ARFs. These transcription factors may in turn enhance the expression of expansins (especially *LmEXPA26*) and aquaporins (especially *LmPIP2-2*), and may ultimately contribute to flower opening. In contrast, in ‘Yulei 1’, the predominance of inactive and irreversibly converted OxIAA may restrict the rapid generation of sufficient active IAA during flowering. As a consequence, the expression of expansins (especially *LmEXPA26*) and aquaporins (especially *LmPIP2-2*) may not be sufficiently elevated, which may limit efficient petal expansion at the appropriate flowering stage and contribute to the persistent bud phenotype.

Flower opening is a complex physiological and biochemical process, and other hormone pathways may also contribute to this process in *L. macranthoides*. Promoter analysis of expansin and aquaporin genes further indicated that these two gene families may also be regulated by other hormone signals, such as ABA and JA. Nevertheless, transcriptomic analysis showed that only auxin-responsive genes were significantly enriched in WT S4 flowers compared with ‘Yulei 1’ S4 flowers. In addition, the differences in auxin-related metabolites between WT S4 flowers and ‘Yulei 1’ S4 flowers further support the close association between auxin and flower opening in *L. macranthoides*. Therefore, this study suggests that flower opening in *L. macranthoides* may be mainly driven by auxin, although the proposed model still requires further experimental validation.

## Discussion

Flowering is an important developmental trait in angiosperms (Doody and Moyroud, 2025). Several studies have demonstrated that expansins and aquaporins play important roles in flower opening (Kong et al., 2018; Ye et al., 2021; Wang et al., 2024a). However, the expansin and aquaporin gene families have not yet been systematically identified in *L. macranthoides*, and their roles during flower opening also remain unclear. In the present study, we identified 34 expansins and 43 aquaporins, the 34 expansins were grouped into 22 EXPAs, 6 EXPBs, 4 EXPLAs, and 2 EXPLBs, and the aquaporins were classified into 12 PIPs, 12 TIPs, 16 NIPs, and 3 SIPs. Within each expansin and aquaporin subfamily, gene structure, protein size, and conserved motifs were largely similar. However, LmEXPB5b and LmEXPA7 differed from other members of their respective subfamilies in gene length, gene structure, and protein size. InterProScan confirmed that both proteins contained the characteristic expansin C-terminal domain/PHL pollen allergen domain and the expansin N-terminal domain/DPBB domain, consistent with typical expansin proteins. They also possessed the conserved motifs of the LmEXPB and LmEXPA subfamilies. According to transcriptome data, *LmEXPB5b* showed nearly no expression in stems, leaves, or flowers, suggesting it may be a pseudogene. In contrast, *LmEXPA7* was expressed in all examined tissues, albeit at low levels, suggesting that LmEXPA7 may represent a non-canonical expansin that could have arisen during *L. macranthoides* evolution via genome duplication or transposon activity (Sun et al., 2021a; Mhiri et al., 2022; Yin et al., 2023; Wang et al., 2024b). For aquaporins, LmPIP1–2 also differed from other LmPIP members in sequence size and gene structure, suggesting that it may be a non-typical aquaporin. However, transcriptome data showed almost no expression of *LmPIP1–2* in stems, leaves, or flowers, indicating that it is also likely a pseudogene.

A detailed analysis of conserved motifs in expansin and aquaporin protein sequences provided additional support for the accuracy of our gene family classification. Expansin proteins typically consist of two major structural domains. The N-terminal domain I is structurally similar to glycoside hydrolase family 45 (GH45) and is also referred to as the double-psi beta-barrel (DPBB) domain (Sun et al., 2021a). This domain represents the functional core of expansins and is responsible for mediating cell wall loosening by promoting the slippage of cellulose microfibrils and disrupting the hydrogen-bonding network within the cell wall. In contrast, the C-terminal domain II resembles the carbohydrate-binding module family 63 (CBM63) and is also known as the pollen allergen domain, which primarily functions in polysaccharide recognition and facilitates the anchoring of expansin proteins to the cell wall (Sun et al., 2021a; Wang et al., 2024b). In the present study, ten conserved motifs were identified in the expansin proteins of *L. macranthoides*. Among them, motifs 1, 4, 5, 7, 8, 9, and 10 were located within the N-terminal domain I, whereas motifs 3 and 6 were distributed within the C-terminal domain II. Interestingly, motif 2 spanned the boundary between the C-terminal region of domain I and the N-terminal region of domain II. In addition, motif 1 contained the characteristic amino acid residues TY … AA, which are important for expansin domain I, while motif 4 included the conserved residue HD, another key structural element of this domain (Sampedro and Cosgrove, 2005). Notably, all ten predicted conserved motifs identified in this study have been reported in previous expansin studies, although minor differences in motif boundaries and sequence conservation have been observed among different species (Sampedro and Cosgrove, 2005; Han et al., 2019b). Differences in conserved amino acids at corresponding motif positions among expansin subfamilies may be associated with functional divergence. For example, motif 2, motif 3, motif 7, and motif 9 were exclusively identified in LmEXPA, suggesting that the conserved amino acids within these motifs may contribute to the catalytic properties of LmEXPA and its interaction with cell wall polysaccharides.

Aquaporin proteins are characterized by a typical structure consisting of six transmembrane α-helices (TM1–TM6), five connecting loops (loops A–E), and cytoplasmic N- and C-termini. Several structural regions, including TM2, TM5, TM6, loop B, loop C, and loop E, are particularly important for aquaporin function, as they contain critical conserved features such as the NPA motifs, the ar/R selectivity filter (aromatic/arginine filter), and Froger’s residues, which collectively determine substrate selectivity and channel permeability (Maurel et al., 2008; Li et al., 2025b). In our analysis of *L. macranthoides* aquaporins, all ten predicted motifs were located within the canonical conserved structural regions of aquaporin proteins. Specifically, motif 11 mainly corresponded to the NPA motif located in loop E, whereas motif 12 represented the NPA motif located in loop B. Motif 14 was primarily located within TM6, motif 15 corresponded to TM3 together with part of loop C, motif 18 was located within the N-terminal region, and motif 20 was mainly distributed within TM1. In addition, motif 17 was specifically detected in the TM1 and the adjacent N-terminal portion of loop A in LmTIPs, while motif 13 showed conserved distribution only in the TM4 and loop D regions of LmPIPs. Interestingly, motif 16 was not only conserved in the TM1 and the N-terminal portion of loop A of LmPIPs, but was also detected in the TM4 and the N-terminal portion of loop D of LmNIPs, LmSIPs, and LmTIPs, suggesting a high degree of sequence similarity between these regions. Similarly, the distribution pattern of motif 15 indicated a relatively high level of sequence conservation between TM2 of LmSIP1–1 and TM5 of LmTIPs. These observations may be related to the internal duplication and structural symmetry of aquaporin proteins, which contain two repeated transmembrane units forming a pseudo-twofold symmetry within the membrane (Maurel et al., 2008; Shivaraj et al., 2017). Similar motif distribution patterns across different transmembrane regions have also been reported in aquaporins from rice (*Oryza sativa*) (Raza et al., 2023), dragon fruit (*Hylocereus undatus*) (Ye et al., 2021), and oat (*Avena sativa*) (Zhou et al., 2023), further supporting the structural conservation of aquaporin proteins across plant species.

In this study, transcriptomic analysis and qRT-PCR results showed that many expansins and aquaporins were upregulated in WT flowers at the S4 stage before flower opening in *L. macranthoides*. Similar expression patterns have also been reported in *Phalaenopsis orchids* (Guan et al., 2025), *Dianthus caryophyllus* (Kong et al., 2018), *Hordeum vulgare* (Li et al., 2021), *Lagerstroemia indica* (Zhang et al., 2025). Flower opening is often closely and positively associated with the expression of expansins, particularly members of the EXPA subfamily, and aquaporins, especially PIP2 genes within the PIP subfamily (Nemoto et al., 2022; Lu et al., 2024; Miao et al., 2024). In our study, *LmEXPA26* showed the highest expression level among expansins in WT flowers at the S4 stage before flower opening, whereas *LmPIP2–2* showed the greatest expression difference between WT and non-opening ‘Yulei 1’ flowers at the S4 stage and also exhibited the highest expression level within the LmPIP subfamily. In addition, both *LmEXPA26* and *LmPIP2–2* showed increased expression during flower opening (S3 to S4 stage), followed by decreased expression after anthesis (S5 stage). These results indicate that the high expression of expansins and aquaporins—represented by *LmEXPA26* and *LmPIP2-2*, respectively—may be associated with petal expansion and flower opening in *L. macranthoides*.

GO enrichment analysis further revealed that genes related to cell-wall and plasma membrane activities were significantly up-regulated in WT S4 flowers compared with ‘Yulei 1’ S4 flowers, supporting the expression data showing strong induction of expansins and aquaporins in WT S4 flowers. Furthermore, transcriptome analysis revealed that only auxin-responsive genes were significantly up-regulated in WT S4 flowers compared with ‘Yulei 1’ S4 flowers. This suggests that auxin signaling may serve as a major driver of flower opening in WT. Similar observations have been made in other species, such as *L. indica* (Zhang et al., 2025), *Phalaenopsis orchids* (Guan et al., 2025), and *R. chinensis* (Han et al., 2019a), where genes related to cell-wall and membrane processes are up-regulated before anthesis, while auxin-related genes are the only hormone-associated genes consistently induced. These findings, together with our results, support a model in which auxin-related genes and genes associated with cell-wall and membrane dynamics—such as expansins and aquaporins—may play essential roles during the pre-anthesis stage to enable flower opening.

Based on this, we quantified auxin-related metabolites in WT and ‘Yulei 1’ S4 flowers. Only MEIAA and OxIAA showed significant differences between the two flower types. MEIAA accumulated to much higher levels in WT S4 flowers compared to ‘Yulei 1’, whereas OxIAA was undetectable in WT S4 flowers but accumulated to high levels in ‘Yulei 1’ S4 flowers. However, the MEIAA content decreased dramatically, whereas the OxIAA content increased sharply when WT flowers were fully opened at the S5 stage. MEIAA is an inactive, reversible storage form of IAA, allowing rapid conversion to active IAA to promote cell growth and expansion (Qin et al., 2005; Zhang et al., 2022). In contrast, OxIAA is an irreversible inactivation product of IAA (Brunoni et al., 2023). In several species, including *Lycium ruthenicum* (Guo et al., 2022), *Litchi chinensis* (Kim et al., 2021), and *Anthurium andraeanum* (Wan et al., 2024), IAA levels continuously rise during flower opening. Therefore, we propose that high MEIAA levels in WT S4 flowers may represent a potential reservoir of IAA, which may contribute to the regulation of IAA concentrations during the rapid transition from S4 to S5 (which occurs within 1–2 hours), while preventing IAA from being metabolized to OxIAA. This mechanism may help maintain sufficient IAA levels at anthesis and may be associated with the flower-opening process. In contrast, the low MEIAA levels and high OxIAA accumulation observed in ‘Yulei 1’ S4 flowers may indicate a reduced capacity to maintain adequate active IAA levels during the flower-opening stage. The increased OxIAA content in fully open flowers (WT S5) may suggest a reduced requirement for maintaining auxin homeostasis, coinciding with the cessation of floral enlargement and the onset of senescence at the S5 stage.

Cis-element analysis of expansin and aquaporin promoters showed that both gene families contain abundant ABA-responsive, JA-responsive, ethylene-responsive, and SA-responsive elements, as well as binding sites for major transcription factor families, including ARF, ERF, GATA, MYB, WRKY, bHLH, bZIP, HD-Zip, and GT, which is consistent with previous reports, suggesting their potential regulation by multiple hormones and transcription factors (Uppuluri et al., 2021; Hu et al., 2024). Moreover, numerous transcription factors corresponding to these cis-regulatory families were up-regulated in WT S4 flowers relative to ‘Yulei 1’ S4 flowers, including 5 ERFs, 4 GTs, 19 MYBs, 14 bHLHs, 9 bZIPs, 2 ARFs, 1 GATA, and 1 HD-Zip. These findings further support the hypothesis that expansins and aquaporins may be co-regulated by multiple hormones and transcription factors. Specifically, ABA-responsive elements and putative binding sites for MYB, bZIP, bHLH, ERF, and GATA transcription factors were identified in the promoters of both *LmEXPA26* and *LmPIP2–2* using the PlantCARE database. In addition, multiple auxin-responsive elements (AuxREs, TGTCNN) were manually detected in both promoters, suggesting that these two genes may be co-regulated by ABA and auxin signaling pathways, as well as their associated transcription factors.

However, the differential accumulation of MEIAA and OxIAA between WT and ‘Yulei 1’ flowers at the S4 and S5 stages, together with the enrichment of auxin signaling pathway-related genes in WT S4 flowers, may provide stronger support for the association of the high expression levels of *LmEXPA26* and *LmPIP2–2* with auxin signaling pathways. In addition, all AuxREs identified in the *LmEXPA26* promoter corresponded to motifs with relatively weak predicted ARF-binding affinity, whereas the *LmPIP2–2* promoter contained one canonical AuxRE motif (TGTCGG) with stronger predicted ARF-binding capacity (Boer et al., 2014; Lieberman-Lazarovich et al., 2019). This pattern is consistent with the higher expression level of *LmPIP2–2* in WT S4 flowers (log_2_(TPM + 1) = 10.22) compared with *LmEXPA26* (log_2_(TPM + 1) = 7.52). Furthermore, MYB-binding sites represented the most abundant predicted transcription factor-binding elements in both promoters. In *R. hybrida*, auxin signaling regulates flower opening through the RhARF2–RhMYB6–RhEXPA8/RhEXPA4/RhPIP2;1 cascade (Chen et al., 2023). In *Osmanthus fragrans*, OfMYB28 promotes flower opening by increasing *OfPIP2* expression and enhancing petal expansion (Lu et al., 2024). These studies provide representative mechanistic examples supporting the possibility that *LmEXPA26* and *LmPIP2–2* are mainly regulated by auxin signaling pathways.

Nevertheless, expansin and aquaporin promoters contain multiple ABA-, JA-, and ethylene-responsive elements, together with other hormone-related cis-elements, the involvement of additional hormonal signals in regulating flower opening in *L. macranthoides* cannot be excluded. Flower opening is a complex physiological process influenced by environmental cues such as light, temperature, water status, and hormones (van Doorn and Kamdee, 2014). Among these hormonal regulators, auxin, ABA, JA, and ethylene have been most extensively implicated in flower opening (Sun et al., 2021b; Salvi and Moyroud, 2025). Auxin appears to play a central role in coordinating petal expansion and movement during flower opening. In *Gentiana rigescens*, auxin exerts antagonistic effects on flower opening under high temperatures and flower closure under low temperatures, indicating that auxin-mediated petal movement is reversible and temperature dependent (Yang et al., 2026). Similar auxin-dependent regulation has also been reported in *Litchi chinensis* (Kim et al., 2021), *Nymphaeales* (waterlily) (Ke et al., 2018), *Fragaria vesca* (Dong et al., 2021), and *Rosa hybrida* (Chen et al., 2023), where auxin promotes flower opening mainly through coordinated activation of expansins and aquaporins, thereby enhancing petal enlargement. In some cases, auxin signaling is further amplified through MYB-mediated transcriptional cascades (Chen et al., 2023; Chopy et al., 2023). This is also consistent with the patterns revealed by the data obtained in the present study. ABA has likewise been recognized as an important regulator of flower opening, particularly under drought-related conditions where accelerated flowering is often promoted (Izawa, 2021). In *Tulipa gesneriana*, ABA has been identified as one of the principal hormones promoting flower opening (Meng et al., 2024). Notably, several studies have shown that ABA-related and auxin-related gene expression often display similar dynamic trends during flower opening (Guo et al., 2022; Singh et al., 2023; Meng et al., 2024), suggesting that these two hormonal pathways may act cooperatively during this process.

Ethylene also contributes positively to flower opening through well-characterized signaling pathways. In *Arabidopsis thaliana*, ethylene receptors such as ETR1 and ERS1 function as transcriptional repressors before ethylene perception, and receptor inactivation after ethylene binding releases downstream signaling that promotes flower opening (van Doorn and Kamdee, 2014). In *Rosa hybrida*, ethylene further promotes flower opening by suppressing the microRNA164–RhNAC100 module through ETHYLENE INSENSITIVE 3 (EIN3), thereby increasing expansin and aquaporin expression (Ma et al., 2008; Pei et al., 2013). Expansins and aquaporins can also be upregulated by auxin (Chen et al., 2023). It suggests potential functional coordination between ethylene and auxin signaling. JA has also been widely associated with petal expansion and flower opening. In *A. thaliana*, reduced expression of JA biosynthetic genes causes delayed petal growth and flower opening (Ishiguro et al., 2001). In *Eustoma grandiflorum*, JA promotes flower opening through the expansin genes *EgEXPA2* and *EgEXPA3*, together with the XTH gene *EgXTH1* (Ochiai et al., 2013). Both JA and auxin can additionally influence flower opening through BPEp-mediated transcriptional regulation, indicating synergistic interactions between these pathways (Salvi and Moyroud, 2025). Exogenous application of methyl jasmonate (MeJA) has also been shown to promote flower opening in *Lonicera japonica* (Li et al., 2025a) and *L. macranthoides* (Pan et al., 2021), further supporting an important role for JA in *Lonicera* flower opening.

At the transcriptional level, ABA commonly regulates downstream targets through bZIP transcription factors (Droge-Laser et al., 2018; Izawa, 2021), whereas JA often acts through MYB and bHLH transcription factors (Furuta et al., 2024; Uji et al., 2024). ERF family transcription factors can be regulated by multiple hormones signaling pathways, such as ethylene, JA, and ABA (Feng et al., 2020). The presence of these transcription factor binding sites in the promoters suggests that expansins and aquaporins (including *LmEXPA26* and *LmPIP2-2*) possess the potential to be regulated by these hormones. However, ABA, JA, and ethylene are also closely associated with post-opening senescence (Sun et al., 2021b; Furuta et al., 2024; Meng et al., 2024), and both JA and ABA frequently exert stronger effects under stress conditions (Izawa, 2021; Vicentini et al., 2023). Therefore, we do not consider that the mechanism by which expansins and aquaporins promote flower opening in *L. macranthoides* is predominantly driven by ABA or ethylene. Although exogenous JA application has been reported to promote flower opening in *L. macranthoides*, the underlying mechanism remains unclear. Moreover, in our transcriptomic data from S4 flowers, JA-responsive genes were not significantly enriched in WT compared with ‘Yulei 1’. By contrast, auxin appears to maintain basal activity during early floral development, when expansins and aquaporins begin to accumulate and promote petal enlargement (Harada et al., 2011; Guan et al., 2025). As flowers approach anthesis, auxin levels often increase transiently, which may further stimulate high expression of expansins and aquaporins and thereby drive rapid petal expansion and movement (Han et al., 2019a; Lu et al., 2024). Auxin levels then typically decline after flower opening (Kim et al., 2021; Guo et al., 2022). In our study, IAA content in WT S4 flowers was higher than in WT S5 flowers, consistent with this general trend. Although the difference between WT S4 and WT S5 was not statistically significant, this may suggest that auxin reaches its maximum accumulation during the partially opened stage rather than during the pre-opening stage, a hypothesis that requires further investigation. Overall, we propose a hypothetical model in which hormonal pathways, particularly auxin signaling, may modulate transcription factors including MYB, bZIP, and ARF, which in turn could influence the expression of expansins (e.g., *LmEXPA26*) and aquaporins (e.g., *LmPIP2-2*), potentially contributing to the flower opening process in *L. macranthoides*.

Flowers of *L. macranthoides* are the major medicinal organs and are rich in chlorogenic acids and saponins, which confer strong medicinal properties, particularly anti-inflammatory, antibacterial, and antiviral activities (Ma et al., 2022). However, under production conditions, flowers of wild-type *L. macranthoides* (WT) rapidly abscise shortly after opening, and important bioactive compounds such as chlorogenic acids decline markedly during this process (Pan et al., 2021; Zeng et al., 2025). As a consequence, flower opening directly reduces harvesting efficiency, yield, and medicinal quality, while simultaneously increasing harvesting costs. In contrast, the closed-bud cultivar ‘Yulei 1’ exhibits a non-opening floral phenotype (Chen et al., 2025), reduced flower abscission, and improved yield and quality. However, because flowers of ‘Yulei 1’ do not open, sexual reproduction is severely restricted, and the cultivar is propagated mainly through vegetative methods such as cutting or grafting in agricultural practice. This long-term asexual propagation has led to reduced genetic diversity, limited varietal improvement, progressive degeneration, weak stress resistance, and frequent occurrence of weak seedlings or seedling death, thereby increasing both cultivation management costs and seedling production costs.

Although previous studies have investigated flower opening mechanisms in *L. macranthoides* and *Lonicera japonica*, their main focus has largely been on floral developmental regulators associated with the ABCDE model (Long et al., 2021; Lin et al., 2023), whereas genes directly involved in petal movement, such as expansins and aquaporins, have received comparatively less attention. Some studies have shown that exogenous jasmonate treatment can promote flower opening in both *L. macranthoides* and *L. japonica* (Pan et al., 2021; Li et al., 2025a). However, both previous transcriptomic studies (Li et al., 2020; Long et al., 2024) and our present results indicate that auxin-related genes are more extensively represented than JA-related genes during flower opening in *L. macranthoides*, while jasmonate is also widely recognized for its important roles in plant stress-response pathways and the regulation of flower senescence (Furuta et al., 2024; Uji et al., 2024). Moreover, the precise molecular mechanism by which JA regulates flower opening in *L. macranthoides* remains unclear. In addition, previous studies combining transcriptomics and metabolomics have attempted to explain flower opening in *L. macranthoides* and *L. japonica*, yet no unified mechanism has been established (Li et al., 2020; Long et al., 2023, 2024). Nevertheless, a finding consistent with the present study is that numerous auxin-related genes were also differentially expressed between unopened flower buds and open flowers in those studies.

In the present study, we propose a hypothetical model of auxin-mediated flower opening involving expansin and aquaporin genes in *L. macranthoides*. This model provides a theoretical basis for regulating flower opening through exogenous hormone application or hormone biosynthesis inhibitors, particularly auxin-related treatments, either to delay flower opening in WT plants or to induce flower opening in ‘Yulei 1’. From a practical perspective, such regulation may help optimize flowering time, reduce cultivation and harvesting costs, and potentially enable hybridization of closed-bud cultivars such as ‘Yulei 1’ under production conditions, thereby accelerating genetic improvement of *L. macranthoides* cultivars. Although the proposed regulatory model still requires further experimental validation, the present study provides a new perspective for understanding the flower opening mechanism of *L. macranthoides*.

## Conclusion

In summary, this study identified 34 expansins and 43 aquaporins in *L. macranthoides*. The expansins were classified into four subfamilies, including 22 EXPAs, 6 EXPBs, 4 EXPLAs, and 2 EXPLBs, while the aquaporins were grouped into four subfamilies comprising 12 PIPs, 12 TIPs, 16 NIPs, and 3 SIPs. Their expression profiles indicated that expansins and aquaporins, particularly *LmEXPA26* and *LmPIP2-2*, were highly expressed at the flower-preparatory stage (S4) in the wild type (WT), but not in ‘Yulei 1’, suggesting a possible association with flower opening. Furthermore, GO enrichment analysis confirmed that genes associated with the cell wall and plasma membrane were upregulated in WT S4 flowers compared with ‘Yulei 1’ S4 flowers, and auxin-related genes were also significantly upregulated in WT S4 flowers. Auxin-related metabolite analysis suggested that a high level of MEIAA may be associated with the flower-opening process in the wild type. Moreover, nine transcription factor families, including MYB, bZIP, and bHLH, were highly expressed in WT S4 flowers. Promoter analysis further revealed numerous cis-regulatory elements in expansin and aquaporin promoters, including ARF-, MYB- and bZIP-binding sites, as well as binding sites for six additional transcription factor families. Overall, these results suggest that expansins and aquaporins, especially *LmEXPA26* and *LmPIP2-2*, may be associated with the flower-opening process in *L. macranthoides*, and that the high level of MEIAA may contribute to flower opening in the wild type. This study clarifies the number and classification of expansin and aquaporin genes and provides new insights into the molecular mechanisms underlying flower opening, which may be useful for germplasm improvement and breeding of *L. macranthoides*.

## Data Availability

The datasets presented in this study can be found in online repositories. The names of the repository/repositories and accession number(s) can be found in the article/**Supplementary Material**.

## Supplementary material

Supplementary Table 1 InterProScan analysis of expansin protein sequences in *Lonicera macranthoides*.

Supplementary Table 2 Protein sequences of the expansin family and the aquaporin family in *L. macranthoides*.

Supplementary Table 3 InterProScan analysis of aquaporin protein sequences *L. macranthoides*.

Supplementary Table 4 Conserved domains, selectivity filters and amino acid residues of aquaporins in *L. macranthoides.*

Supplementary Table 5 Genes location of the expansin family and the aquaporin family in *L. macranthoides*.

Supplementary Table 6 Sequences and validation of primers used in this study.

Supplementary Table 7 Statistical analysis of qRT-PCR data using Student’s t-test.

Supplementary Table 8 Promoter analysis of expansin genes in *L. macranthoides*.

Supplementary Table 9 Promoter analysis of aquaporin genes in *L. macranthoides*.

Supplementary Table 10 Distribution of transcription factor (TF)-binding motifs and AuxREs (auxin response elements) in the promoters of *LmEXPA26* and *LmPIP2-2*.

Supplementary Table 11 Differentially expressed genes (DEGs) and functional annotations in flowers of WT vs. ‘Yulei 1’.

Supplementary Table 12 GO enrichment analysis of significantly up-regulated genes in flowers of WT vs. ‘Yulei 1’.

Supplementary Table 13 GO enrichment analysis of significantly down-regulated genes in flowers of WT vs. ‘Yulei 1’.

Supplementary Table 14 Significantly expressed transcription factors (TF) in flowers of WT vs. ‘Yulei 1’.

Supplementary Table 15 Content of auxin and auxin-associated metabolites in S4 and S5 flowers of WT and ‘Yulei 1’.

## References

[B1] Boer D. R. Freire-Rios A. van den Berg W. A. Saaki T. Manfield I. W. Kepinski S. . (2014). Structural basis for DNA binding specificity by the auxin-dependent ARF transcription factors. Cell. 156, 577–589. doi: 10.1016/j.cell.2013.12.027. PMID: 24485461

[B2] Brunoni F. Pencik A. Zukauskaite A. Ament A. Kopecna M. Collani S. . (2023). Amino acid conjugation of oxIAA is a secondary metabolic regulation involved in auxin homeostasis. New Phytol. 238, 2264–2270. doi: 10.1111/nph.18887. PMID: 36941219

[B3] Chen C. Chen H. Zhang Y. Thomas H. R. Frank M. H. He Y. . (2020). TBtools: An integrative toolkit developed for interactive analyses of big biological data. Mol. Plant 13, 1194–1202. doi: 10.1016/j.molp.2020.06.009. PMID: 32585190

[B4] Chen C. Hussain N. Ma Y. Zuo L. Jiang Y. Sun X. . (2023). The ARF2-MYB6 module mediates auxin-regulated petal expansion in rose. J. Exp. Bot. 74, 4489–4502. doi: 10.1093/jxb/erad173. PMID: 37158672

[B5] Chen Z. Zhuo W. Wang Y. Qi J. Liu L. Lu S. E. . (2025). Mitochondrial genome of Lonicera macranthoides: features, RNA editing, and insights into male sterility. Front. Plant Sci. 15. doi: 10.3389/fpls.2024.1520251. PMID: 39866323 PMC11759266

[B6] Cho L. H. Yoon J. An G. (2017). The control of flowering time by environmental factors. Plant J. 90, 708–719. doi: 10.1111/tpj.13461. PMID: 27995671

[B7] Chopy M. Binaghi M. Cannarozzi G. Halitschke R. Boachon B. Heutink R. . (2023). A single MYB transcription factor with multiple functions during flower development. New Phytol. 239, 2007–2025. doi: 10.1111/nph.19096. PMID: 37394728

[B8] Dong X. Li Y. Guan Y. Wang S. Luo H. Li X. . (2021). Auxin-induced AUXIN RESPONSE FACTOR4 activates APETALA1 and FRUITFULL to promote flowering in woodland strawberry. Hortic. Res. 8, 115. doi: 10.1038/s41438-021-00550-x. PMID: 33931632 PMC8087778

[B9] Doody E. Moyroud E. (2025). Evolution of petal patterning: blooming floral diversity at the microscale. New Phytol. 247, 2538–2556. doi: 10.1111/nph.70370. PMID: 40629857 PMC12371187

[B10] Droge-Laser W. Snoek B. L. Snel B. Weiste C. (2018). The Arabidopsis bZIP transcription factor family-an update. Curr. Opin. Plant Biol. 45, 36–49. doi: 10.1016/j.pbi.2018.05.001. PMID: 29860175

[B11] Feng K. Hou X. L. Xing G. M. Liu J. X. Duan A. Q. Xu Z. S. . (2020). Advances in AP2/ERF super-family transcription factors in plant. Crit. Rev. Biotechnol. 40, 750–776. doi: 10.1080/07388551.2020.1768509. PMID: 32522044

[B12] Furuta Y. Yamamoto H. Hirakawa T. Uemura A. Pelayo M. A. Iimura H. . (2024). Petal abscission is promoted by jasmonic acid-induced autophagy at Arabidopsis petal bases. Nat. Commun. 15, 1098. doi: 10.1038/s41467-024-45371-3. PMID: 38321030 PMC10847506

[B13] Gao B. Zhu L. Liu Z. Li Y. He X. Wu X. . (2023). Chemical composition of honeysuckle (Lonicerae japonicae) extracts and their potential in inhibiting the SARS-CoV-2 spike protein and ACE2 binding, suppressing ACE2, and scavenging radicals. J. Agric. Food. Chem. 71, 6133–6143. doi: 10.1021/acs.jafc.3c00584. PMID: 37021496

[B14] Guan Y. Zhang Q. Zhang T. Li M. Ai Y. Zhai J. . (2025). Transcriptome analysis reveals the mechanisms underlying petal growth during the flower opening process in Phalaenopsis orchids. BMC Plant Biol. 25, 696. doi: 10.1186/s12870-025-06713-5. PMID: 40413394 PMC12102874

[B15] Guo Y. An L. Yu H. Yang M. (2022). Endogenous hormones and biochemical changes during flower development and florescence in the buds and leaves of Lycium ruthenicum Murr. Forests 13, 763. doi: 10.3390/f13050763. PMID: 41725453

[B16] Han Z. Liu Y. Deng X. Liu D. Liu Y. Hu Y. . (2019b). Genome-wide identification and expression analysis of expansin gene family in common wheat (Triticum aestivum L.). BMC Genomics 20, 101. doi: 10.1186/s12864-019-5455-1. PMID: 30709338 PMC6359794

[B17] Han Y. Yong X. Yu J. Cheng T. Wang J. Yang W. . (2019a). Identification of candidate adaxial-abaxial-related genes regulating petal expansion during flower opening in Rosa chinensis “Old Blush. Front. Plant Sci. 10. doi: 10.3389/fpls.2019.01098. PMID: 31552079 PMC6747050

[B18] Harada T. Torii Y. Morita S. Onodera R. Hara Y. Yokoyama R. . (2011). Cloning, characterization, and expression of xyloglucan endotransglucosylase/hydrolase and expansin genes associated with petal growth and development during carnation flower opening. J. Exp. Bot. 62, 815–823. doi: 10.1093/jxb/erq319. PMID: 20959626 PMC3003822

[B19] He Y. Zhao J. Yang B. Sun S. Peng L. Wang Z. (2020). Indole-3-acetate beta-glucosyltransferase OsIAGLU regulates seed vigour through mediating crosstalk between auxin and abscisic acid in rice. Plant Biotechnol. J. 18, 1933–1945. doi: 10.1111/pbi.13353. PMID: 32012429 PMC7415787

[B20] Hu Y. Li Y. Zhu B. Huang W. Chen J. Wang F. . (2024). Genome-wide identification of the expansin gene family in netted melon and their transcriptional responses to fruit peel cracking. Front. Plant Sci. 15. doi: 10.3389/fpls.2024.1332240. PMID: 38322822 PMC10846642

[B21] Ishiguro S. Kawai-Oda A. Ueda J. Nishida I. Okada K. (2001). The DEFECTIVE IN ANTHER DEHISCENCE1 gene encodes a novel phospholipase A1 catalyzing the initial step of jasmonic acid biosynthesis, which synchronizes pollen maturation, anther dehiscence, and flower opening in Arabidopsis. Plant Cell 13, 2191–2209. doi: 10.1105/tpc.010192. PMID: 11595796 PMC139153

[B22] Izawa T. (2021). What is going on with the hormonal control of flowering in plants? Plant J. 105, 431–445. doi: 10.1111/tpj.15036. PMID: 33111430

[B23] Jing T. Wu Y. Yu Y. Li J. Mu X. Xu L. . (2024). Copine proteins are required for brassinosteroid signaling in maize and Arabidopsis. Nat. Commun. 15, 2028. doi: 10.1038/s41467-024-46289-6. PMID: 38459051 PMC10923931

[B24] Johanson U. Karlsson M. Johansson I. Gustavsson S. Sjövall S. Fraysse L. . (2001). The complete set of genes encoding major intrinsic proteins in Arabidopsis provides a framework for a new nomenclature for major intrinsic proteins in plants. Plant Physiol. 126, 1358–1369. doi: 10.1104/pp.126.4.1358. PMID: 11500536 PMC117137

[B25] Katoh K. Rozewicki J. Yamada K. D. (2019). MAFFT online service: multiple sequence alignment, interactive sequence choice and visualization. Brief Bioinform. 20, 1160–1166. doi: 10.1093/bib/bbx108. PMID: 28968734 PMC6781576

[B26] Ke M. Gao Z. Chen J. Qiu Y. Zhang L. Chen X. (2018). Auxin controls circadian flower opening and closure in the waterlily. BMC Plant Biol. 18, 143. doi: 10.1186/s12870-018-1357-7. PMID: 29996787 PMC6042438

[B27] Kende H. Bradford K. Brummell D. Cho H.-T. Cosgrove D. Fleming A. . (2004). Nomenclature for members of the expansin superfamily of genes and proteins. Plant Mol. Biol. 55, 311–314. doi: 10.1007/s11103-004-0158-6. PMID: 15604683

[B28] Kim R. Osako Y. Yamane H. Tao R. Miyagawa H. (2021). Quantitative analysis of auxin metabolites in lychee flowers. Biosci. Biotechnol. Biochem. 85, 467–475. doi: 10.1093/bbb/zbaa083. PMID: 33589897

[B29] Kong W. Bendahmane M. Fu X. (2018). Genome-wide identification and characterization of aquaporins and their role in the flower opening processes in carnation (Dianthus caryophyllus). Molecules 23, 1895. doi: 10.3390/molecules23081895. PMID: 30060619 PMC6222698

[B30] Lanfear R. von Haeseler A. Woodhams M. D. Schrempf D. Chernomor O. Schmidt H. A. . (2020). IQ-TREE 2: New models and efficient methods for phylogenetic inference in the genomic era. Mol. Biol. Evol. 37, 1530–1534. doi: 10.1093/molbev/msaa015. PMID: 32011700 PMC7182206

[B31] Li Y. Gan Y. Qi G. Xu W. Xin T. Huang Y. . (2025a). Transcription factor LjWRKY50 faffects jasmonate-regulated floral bud duration in Lonicera japonica. Plants 14, 2428. doi: 10.3390/plants14152328. PMID: 40805677 PMC12349469

[B32] Li Q. Tong T. Jiang W. Cheng J. Deng F. Wu X. . (2021). Highly conserved evolution of aquaporin PIPs and TIPs confers their crucial contribution to flowering process in plants. Front. Plant Sci. 12. doi: 10.3389/fpls.2021.761713. PMID: 35058944 PMC8764411

[B33] Li Y. Wen S. Li Z. Liu R. Zhang Z. Li Y. . (2025b). The evolution of the aquaporin gene family and drought tolerance mechanisms in green plants. Hortic. Res. 12, uhaf209. doi: 10.1093/hr/uhaf209. PMID: 41180021 PMC12578469

[B34] Li J. Ye C. Chang C. (2020). Comparative transcriptomics analysis revealing flower trichome development during flower development in two Lonicera japonica Thunb. cultivars using RNA-seq. BMC Plant Biol. 20, 341. doi: 10.1186/s12870-020-02546-6. PMID: 32680457 PMC7368687

[B35] Lieberman-Lazarovich M. Yahav C. Israeli A. Efroni I. (2019). Deep conservation of cis-element variants regulating plant hormonal responses. Plant Cell 31, 2559–2572. doi: 10.1105/tpc.19.00129. PMID: 31467248 PMC6881130

[B36] Lin Y. Qi X. Wan Y. Chen Z. Fang H. Liang C. (2023). Genome-wide analysis of the MADS-box gene family in Lonicera japonica and a proposed floral organ identity model. BMC Genomics 24, 447. doi: 10.1186/s12864-023-09509-9. PMID: 37553575 PMC10408238

[B37] Livak K. J. Schmittgen T. D. (2001). Analysis of relative gene expression data using real-time quantitative PCR and the 2(-Delta Delta C(T)) method. Methods 25, 402–408. doi: 10.1006/meth.2001.1262. PMID: 11846609

[B38] Long Y. Q. Liu X. I. A. Zeng J. Li C. A. N. Liu X. D. Zhou R. B. (2021). Cloning and expression of AGL19 gene in two Lonicera macranthoides varieties. J. Genet. 100, 13. doi: 10.1007/s12041-020-01252-4. PMID: 33764335

[B39] Long Y. Zeng J. Liu X. Wang Z. Tong Q. Zhou R. . (2024). Transcriptomic and metabolomic profiling reveals molecular regulatory network involved in flower development and phenotypic changes in two Lonicera macranthoides varieties. 3 Biotech. 14, 1–25. doi: 10.1007/s13205-024-04019-1. PMID: 38855147 PMC11153451

[B40] Long Y. Zeng J. Yang M. Zhou X. Zeng M. Liu C. . (2023). Comparative transcriptome analysis to reveal key ethylene genes involved in a Lonicera macranthoides mutant. Genes Genom. 45, 437–450. doi: 10.1007/s13258-022-01354-6. PMID: 36694039

[B41] Lu X. Kong E. Shen L. Ye Y. Wang Y. Dong B. . (2024). A plasma membrane intrinsic protein gene OfPIP2 involved in promoting petal expansion and drought resistance in Osmanthus fragrans. Int. J. Mol. Sci. 25, 10716. doi: 10.3390/ijms251910716. PMID: 39409047 PMC11477222

[B42] Lv L.-M. Zuo D.-Y. Wang X.-F. Cheng H.-L. Zhang Y.-P. Wang Q.-L. . (2020). Genome-wide identification of the expansin gene family reveals that expansin genes are involved in fibre cell growth in cotton. BMC Plant Biol. 20, 223. doi: 10.1186/s12870-020-02362-y. PMID: 32429837 PMC7236947

[B43] Ma N. Xue J. Li Y. Liu X. Dai F. Jia W. . (2008). Rh-PIP2;1, a rose aquaporin gene, is involved in ethylene-regulated petal expansion. Plant Physiol. 148, 894–907. doi: 10.1104/pp.108.120154. PMID: 18715962 PMC2556823

[B44] Ma A. Zou F. Zhang R. Zhao X. (2022). The effects and underlying mechanisms of medicine and food homologous flowers on the prevention and treatment of related diseases. J. Food Biochem. 46, e14430. doi: 10.1111/jfbc.14430. PMID: 36165435

[B45] Maurel C. Verdoucq L. Luu D.-T. Santoni V. (2008). Plant aquaporins: membrane channels with multiple integrated functions. Annu. Rev. Plant Biol. 59, 595–624. doi: 10.1146/annurev.arplant.59.032607.092734. PMID: 18444909

[B46] Meng L. Yang H. La Y. Wu Y. Ye T. Wang Y. . (2024). Transcriptional modules and hormonal metabolic pathways reveal the critical role of TgHB12-like in the regulation of flower opening and petal senescence in Tulipa gesneriana. Horticulture Adv. 2, 18. doi: 10.1007/s44281-024-00031-w. PMID: 41933263

[B47] Mhiri C. Borges F. Grandbastien M. A. (2022). Specificities and dynamics of transposable elements in land plants. Biol. (Basel) 11, 488. doi: 10.3390/biology11040488. PMID: 35453688 PMC9033089

[B48] Miao Y. Li W. Zhu H. Wang Y. Fang Q. Xiao Z. . (2024). The roles of OfEXPA2 and OfEXPA4 on petal cell expansion during flower opening in Osmanthus fragrans. Sci. Hortic. 338, 113720. doi: 10.1016/j.scienta.2024.113720. PMID: 41936479

[B49] Nemoto K. Niinae T. Goto F. Sugiyama N. Watanabe A. Shimizu M. . (2022). Calcium-dependent protein kinase 16 phosphorylates and activates the aquaporin PIP2;2 to regulate reversible flower opening in Gentiana scabra. Plant Cell 34, 2652–2670. doi: 10.1093/plcell/koac120. PMID: 35441691 PMC9252468

[B50] Ochiai M. Matsumoto S. Yamada K. (2013). Methyl jasmonate treatment promotes flower opening of cut Eustoma by inducing cell wall loosening proteins in petals. Postharvest Biol. Technol. 82, 1–5. doi: 10.1016/j.postharvbio.2013.02.018. PMID: 41936479

[B51] Pan Y. Zhao X. Wu X. Wang Y. Tan J. Chen D. (2021). Transcriptomic and metabolomic analyses provide insights into the biosynthesis of chlorogenic acids in Lonicera macranthoides Hand.-Mazz. PloS One 16, e0251390. doi: 10.1371/journal.pone.0251390. PMID: 34038434 PMC8153468

[B52] Pei H. Ma N. Tian J. Luo J. Chen J. Li J. . (2013). An NAC transcription factor controls ethylene-regulated cell expansion in flower petals. Plant Physiol. 163, 775–791. doi: 10.1104/pp.113.223388. PMID: 23933991 PMC3793057

[B53] Qin C. Du T. Zhang R. Wang Q. Liu Y. Wang T. . (2023). Integrated transcriptome, metabolome and phytohormone analysis reveals developmental differences between the first and secondary flowering in Castanea mollissima. Front. Plant Sci. 14. doi: 10.3389/fpls.2023.1145418. PMID: 37008486 PMC10060901

[B54] Qin G. Gu H. Zhao Y. Ma Z. Shi G. Yang Y. . (2005). An indole-3-acetic acid carboxyl methyltransferase regulates Arabidopsis leaf development. Plant Cell 17, 2693–2704. doi: 10.1105/tpc.105.034959. PMID: 16169896 PMC1242266

[B55] Raza Q. Rashid M. A. R. Waqas M. Ali Z. Rana I. A. Khan S. H. . (2023). Genomic diversity of aquaporins across genus Oryza provides a rich genetic resource for development of climate resilient rice cultivars. BMC Plant Biol. 23, 172. doi: 10.1186/s12870-023-04151-9. PMID: 37003962 PMC10064747

[B56] Salvi E. Moyroud E. (2025). Building beauty: Understanding how hormone signaling regulates petal patterning and morphogenesis. Plant J. 121, e70101. doi: 10.1111/tpj.70101. PMID: 40106266 PMC11922171

[B57] Sampedro J. Cosgrove D. J. (2005). The expansin superfamily. Genome Biol. 6, 242. doi: 10.1186/gb-2005-6-12-242. PMID: 16356276 PMC1414085

[B58] Shivaraj S. M. Deshmukh R. K. Rai R. Belanger R. Agrawal P. K. Dash P. K. (2017). Genome-wide identification, characterization, and expression profile of aquaporin gene family in flax (Linum usitatissimum). Sci. Rep. 7, 46137. doi: 10.1038/srep46137. PMID: 28447607 PMC5406838

[B59] Singh D. Sharma S. Jose-Santhi J. Kalia D. Singh R. K. (2023). Hormones regulate the flowering process in saffron differently depending on the developmental stage. Front. Plant Sci. 14. doi: 10.3389/fpls.2023.1107172. PMID: 36968363 PMC10034077

[B60] Sun X. Qin M. Yu Q. Huang Z. Xiao Y. Li Y. . (2021b). Molecular understanding of postharvest flower opening and senescence. Mol. Horticulture 1, 7. doi: 10.1186/s43897-021-00015-8. PMID: 37789453 PMC10514961

[B61] Sun W. Yu H. Liu M. Ma Z. Chen H. (2021a). Evolutionary research on the expansin protein family during the plant transition to land provides new insights into the development of Tartary buckwheat fruit. BMC Genomics 22, 252. doi: 10.1186/s12864-021-07562-w. PMID: 33836656 PMC8034093

[B62] Tang N. Cao Z. Yang C. Ran D. Wu P. Gao H. . (2021). A R2R3-MYB transcriptional activator LmMYB15 regulates chlorogenic acid biosynthesis and phenylpropanoid metabolism in Lonicera macranthoides. Plant Sci. 308, 110924. doi: 10.1016/j.plantsci.2021.110924. PMID: 34034872

[B63] Uji Y. Suzuki G. Fujii Y. Kashihara K. Yamada S. Gomi K. (2024). Jasmonic acid (JA)-mediating MYB transcription factor1, JMTF1, coordinates the balance between JA and auxin signalling in the rice defence response. Physiol. Plant 176, e14257. doi: 10.1111/ppl.14257. PMID: 38504376

[B64] Uppuluri L. S. Motukuri S. R. K. Kumar D. (2021). Genome-wide identification, characterization of aquaporin gene family and understanding aquaporin transport system in hot pepper (Capsicum annuum L.). Sci. Hortic. 286, 110206. doi: 10.1016/j.scienta.2021.110206. PMID: 41936479

[B65] van Doorn W. G. Kamdee C. (2014). Flower opening and closure: an update. J. Exp. Bot. 65, 5749–5757. doi: 10.1093/jxb/eru327. PMID: 25135521

[B66] Vicentini G. Biancucci M. Mineri L. Chirivi D. Giaume F. Miao Y. . (2023). Environmental control of rice flowering time. Plant Commun. 4, 100610. doi: 10.1016/j.xplc.2023.100610. PMID: 37147799 PMC10504588

[B67] Wan X. Zou L. H. Pan X. Ge Y. Jin L. Cao Q. . (2024). Auxin and carbohydrate control flower bud development in Anthurium andraeanum during early stage of sexual reproduction. BMC Plant Biol. 24, 159. doi: 10.1186/s12870-024-04869-0. PMID: 38429715 PMC10908059

[B68] Wang Z. Cao J. Lin N. Li J. Wang Y. Liu W. . (2024b). Origin, evolution, and diversification of the expansin family in plants. Int. J. Mol. Sci. 25, 11814. doi: 10.3390/ijms252111814. PMID: 39519364 PMC11547041

[B69] Wang Y. Qin M. Zhang G. Lu J. Zhang C. Ma N. . (2024a). Transcription factor RhRAP2.4L orchestrates cell proliferation and expansion to control petal size in rose. Plant Physiol. 194, 2338–2353. doi: 10.1093/plphys/kiad657. PMID: 38084893

[B70] Yang L. Chen D. Dong Y. Chen S. Wang W. Yu C. . (2026). The Gentiana rigescens genome and transcriptome for the investigation of flower repetitive blooming. BMC Biol. 24, 38. doi: 10.1186/s12915-025-02483-6. PMID: 41388462 PMC12895662

[B71] Yang R. Ma Y. Yang Z. Pu Y. Liu M. Du J. . (2024). Knockdown of beta-conglycinin alpha’ and alpha subunits alters seed protein composition and improves salt tolerance in soybean. Plant J. 120, 1488–1507. doi: 10.1111/tpj.17062. PMID: 39383405

[B72] Ye X. Gao Y. Chen C. Xie F. Hua Q. Zhang Z. . (2021). Genome-wide identification of aquaporin gene family in Pitaya reveals an HuNIP6;1 involved in flowering process. Int. J. Mol. Sci. 22, 7689. doi: 10.3390/ijms22147689. PMID: 34299311 PMC8306030

[B73] Yin X. Xiang Y. Huang F. Q. Chen Y. Ding H. Du J. . (2023). Comparative genomics of the medicinal plants Lonicera macranthoides and L. japonica provides insight into genus genome evolution and hederagenin‐based saponin biosynthesis. Plant Biotechnol. J. 21, 2209–2223. doi: 10.1111/pbi.14123. PMID: 37449344 PMC10579715

[B74] Yu W. Dai Y. Chen J. Liang A. Wu Y. Suo Q. . (2024). Upregulation of the glycine-rich protein-encoding gene GhGRPL enhances plant tolerance to abiotic and biotic stressors by promoting secondary cell wall development. J. Integr. Agric. 23, 3311–3327. doi: 10.1016/j.jia.2024.05.025. PMID: 41936479

[B75] Zeng J. Long Y. Q. Zhu J. Y. Fu X. S. Zhang J. Y. He J. W. . (2025). Accumulation differences of high-value ingredients in different phenotype Lonicera macranthoides: insights from integrative metabolome and transcriptome analyses. Front. Plant Sci. 16. doi: 10.3389/fpls.2025.1533263. PMID: 40104033 PMC11913843

[B76] Zhang F. Li C. Qu X. Liu J. Yu Z. Wang J. . (2022). A feedback regulation between ARF7-mediated auxin signaling and auxin homeostasis involving MES17 affects plant gravitropism. J. Integr. Plant Biol. 64, 1339–1351. doi: 10.1111/jipb.13268. PMID: 35475598

[B77] Zhang G. Zhao Y. Wu Z. Li Z. Zong J. Yang L. . (2025). Deciphering the genetic basis of flower opening in Lagerstroemia indica: transcriptomic and functional insights into expansin-mediated petal expansion. Ornamental Plant Res. 5, e018. doi: 10.48130/opr-0025-0013

[B78] Zhou X. Yi D. Ma L. Wang X. (2023). Genome-wide analysis and expression of the aquaporin gene family in Avena sativa L. Front. Plant Sci. 14. doi: 10.3389/fpls.2023.1305299. PMID: 38312362 PMC10836146

